# Shedding Light on Chemically Mediated Tri-Trophic Interactions: A ^1^H-NMR Network Approach to Identify Compound Structural Features and Associated Biological Activity

**DOI:** 10.3389/fpls.2018.01155

**Published:** 2018-08-17

**Authors:** Lora A. Richards, Celso Oliveira, Lee A. Dyer, Arran Rumbaugh, Federico Urbano-Muñoz, Ian S. Wallace, Craig D. Dodson, Christopher S. Jeffrey

**Affiliations:** ^1^Department of Biology, University of Nevada, Reno, NV, United States; ^2^Department of Chemistry, University of Nevada, Reno, NV, United States; ^3^Department of Biochemistry and Molecular Biology, University of Nevada, Reno, NV, United States

**Keywords:** NMR, *Piper*, network analysis, chemical ecology, multi-tropic interactions

## Abstract

Diverse mixtures of plant natural products play an important role in plant-herbivore-parasitoid interactions. In the pursuit of understanding these chemically-mediated interactions, we are often faced with the challenge of determining ecologically and biologically relevant compounds present in complex phytochemical mixtures. Using a network-based approach, we analyzed binned ^1^H-NMR data from 196 prepared mixtures of commonly studied secondary metabolites including alkaloids, amides, terpenes, iridoid glycosides, saponins, phenylpropanoids, flavonoids and phytosterols. The mixtures included multiple dimensions of chemical diversity, including molecular complexity, mixture complexity and differences in relative compound concentrations. This approach yielded modules of co-occurring chemical shifts that were correlated with specific compounds or common structural features shared across compounds. This approach was then applied to crude phytochemical extracts of 31 species in the phytochemically diverse tropical plant genus *Piper* (Piperaceae). Combining the activity of crude plant extracts in an array of bioassays with our ^1^H-NMR network approach, we identified a potential prenylated benzoic acid from these mixtures that exhibits antifungal properties and identified small structural differences that were potentially responsible for antifungal activity. In an intraspecific analysis of individual *Piper kelleyi* plants, we also found ontogenetic differences in chemistry that may affect natural plant enemies. In sum, this approach allowed us to characterize mixtures as useful network modules and to combine chemical and ecological datasets to identify biologically important compounds from crude extracts.

## Introduction

Over evolutionary time, selective forces have shaped the unique secondary metabolite profile in every plant species on Earth. These complex chemical mixtures function as defenses against multiple natural enemies, including herbivores and pathogens (Fraenkel, 1959), and as the specifics of ecological communities that affect plant species differ, so does the defensive chemistry. Since Ehrlich and Raven's (Ehrlich and Raven, 1964) influential study proposing a coevolutionary arms race between plant and herbivore taxa linked to codiversification and increased specialization, many studies have focused on understanding the causes and consequences of phytochemical composition on plant-insect interactions. Due to the limitations in methods used to chemically profile plant secondary metabolites, most of these studies have focused on a few well-studied plant species and specific classes of compounds. While these studies have contributed to our understanding of specific interactions mechanisms, we are still limited in our ability to address larger hypotheses that describe patterns found across taxa.

The traditional approach of natural products isolation is time intensive and often results in inactive or redundant compounds. This is not surprising considering the increasing number of studies demonstrating that natural products often act synergistically (Richards et al., 2016), additively, or antagonistically (Diawara et al., 1993). Therefore, quantifying patterns of phytochemical variation is a fundamental goal of natural products chemistry as well as chemical ecology. The ability to link specific phytochemical features to a biological or ecological response can be a useful method for streamlining the identification of relevant compounds and can be used as an approach to dereplication (Kurita et al., 2015). With the recent development of metabolomics approaches, the process of characterizing complex natural product mixtures has become more efficient. These established approaches are well suited for identifying phytochemical differences between individuals of the same species, where there are many shared metabolites across samples, but there are still limitations when metabolomics comparisons are made between different species with limited overlap in their chemical composition. Additionally, statistical analysis of spectral data from complex mixtures have focused on extracts of well-defined systems such as *E. coli* (Koek et al., 2006; Winder et al., 2008), urine (Weljie et al., 2006), blood (Zelena et al., 2009), and crops (Catchpole et al., 2005; Dixon et al., 2006; Witt et al., 2012), but there are additional challenges to studying non-model organisms that have not been thoroughly chemically characterized.

Metabolomics based approaches use two main spectroscopic methods for chemical analysis; mass spectrometry (MS) and nuclear magnetic resonance (NMR) (Sumner et al., 2003; Barding et al., 2012). Arguably, utilizing a combination of both spectroscopic techniques will provide complementary information on the full metabolic profile (Barding et al., 2013; Wolfender et al., 2015). While MS is the most commonly used, due to the coupling with chromatography, accessibility to instruments, and sensitivity, NMR has several advantages over MS analyses that are important when comparing multiple species that do not have similar chemical profiles (Richards et al., 2015). For example, NMR is less dependent upon the chemical properties of specific compound classes and can detect a broader range of compounds (i.e., volatiles, non-volatiles, and a range in polarity) (Kim et al., 2011; Leiss et al., 2011) in a single analysis. In addition, NMR data are highly reproducible, quantitative, and provide structural resolution as well as facile peak alignment across spectra. Complementary to these spectroscopic techniques, appropriate multivariate analyses can expedite the process of data-mining from metabolomics datasets, for which ordination techniques such as PCA (Principal Component Analysis), PLS-DA (Partial Least Squares Discriminant Analysis) and NMDS (Non-metric Multidimensional Scaling) have been primarily used. These techniques focus on identifying the smallest number of variables (peaks, chemical shifts in NMR and retention times/molar masses in MS) that account for the largest proportion of variation between spectra. These methods are well suited for quantifying dramatic variation between chemically similar samples, such as plants of different cultivars, but do not perform well when identifying the subtle differences necessary for applications in quantitative interspecies comparisons where the number of distinguishing variables is far more expansive (Covington et al., 2017). Recent developments in MS data analysis provided a powerful means to build molecular networks that connect samples based on chemotypic similarity (Garg et al., 2015). These networks can be used in combination with pure compound libraries or seed molecules to facilitate compound dereplication and identify novel compounds in a profile (Watrous et al., 2012). With a similar innovative approach, we tested the application of weighted network analysis to identify compounds in mixtures of secondary metabolites from ^1^H-NMR data and link chemical composition to bioactivity. In a weighted network the connection between two nodes (edge) is quantified as a number between 0 and 1. In a weighted network of ^1^H-NMR data, the nodes represent proton resonances and the weight of the edge is calculated as an adjacency value based on the correlation of resonances across the spectra. Modules, groups of highly correlated nodes, are identified by hierarchical clustering. We predicted that these modules would be comprised of resonances of a single compound or of resonances representing shared structural elements across multiple compounds, as a single molecule is represented by multiple resonances. One of the important features of the approach presented here is the ability to calculate the weight of the module (collection of resonances) for each spectra. This continuous variable provides a relative weight of each module on each individual spectra and can then be used to correlate modules to the biological or ecological data of the sample. As this was the first application of this approach to ^1^H-NMR data, we first set out to establish that the modules identified correspond to protons related to the same compound or shared chemical structure. Therefore, we utilized a controlled experiment with prepared mixtures of natural products, manipulated mixture complexity and then validated the results. We then applied the analysis to crude extracts of plants in the phytochemically diverse genus *Piper* (Piperaceae).

## Materials and methods

### Prepared mixture application

#### Samples

We selected 29 pure natural product compounds, either from commercial sources or isolated from plant extracts (Table 1), to simulate the chemical complexity of the *Piper* genus. Although this set of compounds includes only a small portion of the thousands of known natural products produced by plants, it represents the major groups of small molecules implicated in plant defense (Rosenthal, 1991), including terpenoids, flavonoids, phenylpropanoids, furanocoumarins, amides, and alkaloids (Table 1). The compounds were initially dissolved in CD_3_OD (Cambridge Isotope Laboratories, Inc.), (99.8%) containing 0.05% TMS to prepare stock solutions at a concentration of 10 mg/mL. While methanol is not an ideal solvent for ^1^H-NMR analysis due to the two wide residual peaks in regions that can overlap with sample peaks, it offered the best solubility to the set of compounds and is a practical solvent used to extract a broad range of metabolites in untargeted experiments (Martin et al., 2014). The prepared solutions were then combined into mixtures consisting of three to four different compounds to a total of 1 mL per mixture. These combinations were designed to capture multiple attributes of crude phytochemical extracts found in nature: “Intraclass” mixtures included 21 combinations of three compounds within same metabolic group at a mass ratio of 3:1:1; “Interclass” mixtures included 97 combinations of three compounds from two different metabolic groups at a mass ratio of 3:1:1; and 78 “4-component” mixtures included four compounds from three different compound classes at a mass ratio of 2:1:1:1, yielding a combination for which the major component was at a lower relative concentration and compound diversity was higher. Both interclass and 4-component mixtures contained two compounds of the same metabolic group in order to simulate the general observation from natural plant extracts that compounds of the same biosynthetic pathway tend to co-occur (Gershenzon et al., 2012). In addition to compounds of specific metabolic groups, some of the interclass and 4-component mixtures also contained eicosanol, which simulates the effect of long chain fatty acids or other aliphatic compounds commonly present in plant extracts and yields an increase in peak overlap in the upfield region of the ^1^H-NMR spectrum (δ 0.5–2).

**Table 1:** Relative accuracy of the three analyses used to identify proton resonances associated with a specific compound.

		**Relative accuracy**		
**Compounds**		**Intraclass**	**Interclass**	**4 compounds**
Alkaloids	Brucine	0.33	0.35	0.35
	Boldine	0.58	0.80	0.68
	Crotaline	0.32	0.57	–
	Caffeine	–	0.19	0.19
Amides	Alkene amide	0.64	0.56	0.44
	Piplartine	0.88	0.63	0.72
	Pipleroxide	0.58	0.48	0.38
Iridoid glycosides	Aucubin	–	0.34	–
	Catalposide	0.28	0.28	0.41
	Catalpol	0.23	0.36	0.36
Cardiac glycosides	Digitoxin	–	0.21	0.25
Furanocoumarins	Bergapten	–	1.00	0.88
	Imperatorin	–	0.88	–
	Xanthotoxin	–	0.67	0.83
Flavonoids	Rutin	0.54	0.45	0.54
Isoflavonoid	Daidzein	0.95	0.95	0.00
	Daidzin	0.58	0.78	0.25
	Genistein	–	0.60	0.80
Terpenoids	Carene	0.86	0.86	0.86
	Phytol	0.53	0.53	0.72
	Nerolidol	0.69	0.94	0.59
Triterpeinoid saponins	Escin	0.26	0.17	0.31
Saponin	Diosgenin	0.56	0.49	–
	Oleanolic acid	0.56	0.46	–
Phenylpropenoids	Eugenol	0.43	0.86	0.71
	Resveratrol	1.00	0.83	0.83
	Prenylated benzoic acid	0.48	0.33	–
Phytosterols	Sitosterol	–	0.77	0.44
	Stigmasterol	–	0.50	0.38

*The values shown for each compound represent the highest correlation calculated for a module. The compound structural features and the specific chemical shifts identified by each module are listed in the SI (Tables S1–S3)*.

#### ^1^H-NMR analysis

The total collection of over 196 prepared mixtures was analyzed by ^1^H-NMR spectroscopy using a Varian 400-MR (400 MHz) spectrometer, with 64 scans per spectrum (Table S1 for experimental details). Additionally, ^1^H-NMR spectra were independently acquired for the pure compounds in order to support the complete compound-peak assignments and validate the recognition of molecular patterns in the statistical and network analyses. In cases of severe peak overlap, such as with glycosylated groups and steroidal compounds, two-dimensional techniques (COSY, HSQC, and HMBC) were used exclusively to assist in resolving peak assignments for the pure compounds. The software MNova (version 10.0, Mestrelab Research, Santiago de Compostela, Spain) was used in spectral treatment and data extraction. Each spectrum was individually aligned by the residual methanol peak (septet, δ_H_ 3.31), collectively phase-corrected (global method) and baseline-corrected (polynomial fit), then binned in 0.04 ppm bins from 12 to 0.5 ppm (bins integrated by average sum) (Verpoorte et al., 2007). After binning the data, the solvent peak residuals were removed, the spectra were normalized to a total area of 100 units, and the resulting data were exported into a .csv file to be used in network analyses.

#### Statistical analysis

Applying a weighted network approach (Zhang and Horvath, 2005; Horvath, 2011), we analyzed spectral data to build a network in which the nodes were binned chemical shifts from ^1^H-NMR and the edges were determined based on the correlations between the chemical shifts. Therefore, chemical shifts that co-varied across samples are more connected. We organized these highly connected chemical shifts into modules. All the network analyses were performed in the statistical software R version 3.2.3 (R Delevopment Core Team, 2015) using the WGCNA package (Langfelder and Horvath, 2008, 2012). A soft threshold (β) was used in a power transformation of the correlation coefficient to determine node adjacency, or connection strength. The software package allows the analysis of signed or unsigned correlation networks. In an unsigned network, the adjacency (*a*) between two nodes is calculated as $a_{ij}=|cor{\left(x_{i\;},\;\;x_{j}\right)|}^{\beta }$, where an adjacency value is calculated for both positive and negative correlation coefficients. In comparison, in a signed network, adjacency is calculated as $a_{ij}=|0.5+0.5\times cor{\left(x_{i\;},\;\;x_{j}\right)|}^{\beta }$, where an adjacency value is only calculated for positive correlation coefficients (Zhang and Horvath, 2005; Horvath, 2011). While this distinction is useful for analyzing gene-expression data (Zhang and Horvath, 2005) to identify genes that upregulate together (only positive correlations between genes) and not combinations of genes where one gene is expressed when another gene is downregulated (negative correlation between genes), both signed and unsigned networks identified the same network modules for all ^1^H-NMR datasets presented here, indicating that the nodes were only positively correlated to each other. This was not surprising due to the nature of the dataset. We would expect the correlation between proton resonances of a compound to be positive, since they are interdependent and the presence one peak does not limit the potential presence of another. Therefore, all the networks were analyzed as unsigned. We used the lowest value for β that produced the most scale-free topology in the network. For binned spectral data, this ensured separation of the baseline from meaningful peaks. Next, a correlation network topological overlap matrix was calculated, and an average linkage hierarchical cluster analysis was used to identify modules within the network (defined as clusters of highly connected nodes, chemical shifts). Applying the *blockwiseModules* function in the WGCNA package, a minimum module size was defined as three chemical shifts and merged modules were correlated by 0.75 (parameter mergeCutHeight = 0.25). The modules were assigned an arbitrary color code to aid in network visualization. Through singular value decomposition, an eigenvector for each module was used to calculate a module eigenvalue for each spectrum. Calculating an eigenvector, which is analagous to the first principal component of a Principal Component Analysis, allowed us to examine correlations between modules and the individual compound concentrations in the artificial mixtures. The networks were visualized in Cytoscape (Shannon et al., 2003).

To validate the efficacy of our NMR-based approach and verify the appropriateness of compound assignments to modules, we examined correlations of module eigenvalues with concentrations of individual compounds in the mixtures, but considering only module-compound combinations with a significant Pearson's correlation (*p* ≤ 0.05). Chemical shifts in each module were mapped to the structure of the corresponding molecules, according to the ^1^H-NMR spectra of mixtures and pure compounds (Tables S2–S4). For each compound, we determined the number of maximum distinguishable signals based on a visual inspection of their individual spectra, where peaks within 0.05 ppm are considered part of the same signal. We then determined what proportion of those signals are recognized by a module, accordingly to the module's representative chemical shifts. Exact chemical shift matches were assigned an accuracy value of 1, while module chemical shifts within 0.1 ppm of a recognized compound peak were valued as 0.75. The relative accuracy of a module to represent a compound was defined as the ratio of the sum of matches to the maximum number of distinguishable compound peaks.

### Interspecific *Piper* application

#### Samples

We validated the approach with crude plant extracts from 31 species of the phytochemically diverse tropical genus *Piper* (Piperaceae) (Dyer and Palmer, 2004). In this cross taxa analysis, we collected the most recently expanded leaves from 31 different *Piper* species at La Selva Biological Station in Costa Rica, Heredia Province (10°25′ N, 84°00′ W, 50 m). Leaves from multiple individuals were pooled for each species. All samples were dried in an air-conditioned laboratory, ground with mortar and pestle to a fine powder, and 2 g of this powder was transferred to a screw cap test tube and combined with 10 mL of methanol. The samples were sonicated for 10 min and filtered to separate the leaf material from the supernatant. This step was repeated a second time, and the supernatants were combined and transferred to a pre-weighed 20 mL scintillation vials. The solvent was removed under reduced pressure at 30°C and prepared for NMR analysis.

#### ^1^H-NMR analysis

For each sample, 15 mg of plant extract was dissolved in 1 ml of deuterated methanol and analyzed by ^1^H-NMR spectroscopy as described for the prepared mixtures, but in this case, 128 scans were collected per spectrum in order to maximize the detection of minor compounds in the crude extracts. Spectra treatment and data organization was performed according to the protocol previously described for the prepared mixtures.

#### Bioassays

Extracts from 31 species of *Piper* were assayed in four different panels: (1) an insect bioassay using the generalist herbivore *Spodoptera exigua* (Noctuidae, Lepidoptera); (2) a bacterial growth assay using *Escherichia coli* (Enterobacteriaceae); (3) a yeast growth assay with *Saccharomyces cerevisiae* (Saccharomycetaceae); and (4) a growth assay using the plant *Arabidopsis thaliana* (Brassicaceae).

*Spodoptera exigua* eggs were purchased through Benzon Research (Carlisle, PA) and a laboratory colony was maintained on beet armyworm artificial diet (Southland Products, Lake Village, AR). Experimental diets were made by replacing the 30% of the artificial diet dry ingredients with dry ground leaf material of *Piper*. Second instar *S. exigua* larvae where weighed and placed in individual cups with experimental diet, with a total of 20 larvae per *Piper* species treatment. Larvae were checked daily to monitor survival, molting and resupply of diet as needed. We recorded survival, development time and pupal mass.

*Escherichia coli* strain DH5α cells were grown on Luria Broth (LB) solid media and incubated at 37°C for 16 hr. Single colonies were then used to inoculate 10 mL LB liquid cultures, which were incubated at 37°C for 16 h with shaking. Aliquots of the saturated cultures were diluted 100-fold in LB liquid medium. *Piper* extracts were dissolved in methanol at a standardized concentration of 80 mg/mL, and test extracts were added to the diluted *E. coli* cultures at a concentration of 80 μg/mL. Two hundred microliter samples were arrayed into individual wells of a sterile 96-well plate and sealed with clear adhesive film. The plate was placed in a SpectraMax M2^e^ 96-well plate reader (Molecular Devices, Sunnyvale, CA) equilibrated at 37°C. The absorbance at 600 nm (OD_600_) was measured every 5 min for 12 h with an initial shake time of 5 s and 3 s shake prior to each reading.

*Saccharomyces cerevisiae* growth curves were measured in a similar manner. *S. cerevisiae* S288c cells were plated on YPD media (2% [w/v] peptone, 1% [w/v] yeast extract, 2% [w/v] glucose) and incubated for 2 days at 30°C. A single colony was used to inoculate a 10 mL YPD culture, which was incubated at 30 °C for 18 h with shaking. Saturated *S. cerevisiae* cultures were diluted 100-fold into liquid YPD, and extracts were diluted into these cultures as described above. Samples were arrayed into 96-well plated and sealed with adhesive film. A sterile needle was used to puncture a small hole in the adhesive film above each well to prevent gas buildup. The resulting plate was assayed in a SpectraMax M2^e^ 96-well plate reader as described above with OD_600_ readings taken at 5 min intervals for 18 h with 30 s of shaking before each reading.

*Arabidopsis thaliana* Col-0 seeds were surface sterilized with seed cleaning solution (3% [v/v] sodium hypochlorite, 0.1% [w/v] sodium dodecylsulfate) for 20 min at 25 °C. The seed cleaning solution was removed and seeds were washed five times in sterile water. Seeds were resuspended in sterile water, incubated at 4 °C for 48 h, then plated on MS-agar media (1/2X Murashige and Skoog salts, MES-KOH pH 5.7, 1% [w/v] sucrose, 1% [w/v] phytoagar) with or without the addition of *Piper* extracts at a final concentration of 80 μg/mL. Plants were grown vertically in a growth chamber at 22°C with constant light for 7 days. The roots of each seedling were straightened, and the resulting plants were imaged on a flatbed scanner. Root lengths were measured using ImageJ (imageJ.nih.gov/ij/).

#### Statistical analysis

To verify the approach using extracts from the cross taxa samples, we performed a network analysis using ^1^H-NMR data of the 31 *Piper* extracts along with spectra from the prepared mixtures to seed the analysis with known structures. *Piper* species produce many classes of secondary metabolites, therefore we included a training set of spectra of 71 prepared mixtures in the analysis which represented 21 different compounds that varied in concentration. We excluded compound classes that have not been recorded in *Piper*, including furanocoumarins, saponins and irioid glycosides (except catalpol which was in mixtures with other compounds). After modules of co-occurring chemical shifts across the extracts were identified, we calculated the correlations between module eigenvalues for each sample and their bioassay values. We then identified the compounds from the seeded prepared mixtures that had a significant correlation to specific module eigenvalues, which allowed us to make assumptions regarding the identity of bioactive molecules in the extracts.

### Intraspecific *Piper* application

#### Samples

For intraspecies analysis, leaf samples were obtained from multiple individuals of a single species, *P. kelleyi*, from Yanayacu Biological Station, Napo Province, Ecuador (0°36′ S, 77°53′ W, 2080- m). We collected the most recently expanded leaves, and when available, young leaves from plants at different developmental stages. Key morphological features of *Piper* are alternate leaves and jointed stems with enlarged nodes; the number of nodes on an individual plant indicates the total number of leaves produced, which is correlated with plant age. Therefore, we collected leaves from three age categories, adult (>25 nodes, *N* = 12), “saplings” (< 20 nodes, *N* = 18) and “seedlings” (or plantlets, < 10 nodes, *N* = 17). Samples were then dried and prepared according to the protocol previously described.

#### ^1^H-NMR analysis

The spectroscopic analysis of extracts of *P. kelleyi* follows that described for the interspecific study of *Piper* species.

#### Statistical analyses

For validating its applicability to data from within a species, we performed the network analysis on the ^1^H-NMR data from *P. kelleyi* extracts. We followed the analysis with a Multiple Analysis of Variance (MANOVA) using module eigenvalues as dependent variables and developmental stage (adult, seedling, and sapling) and leaf age (young vs. fully expanded) as predictor variables. Modules that demonstrated a significant main effect of developmental stage were analyzed using Tukey's HSD *post-hoc* tests to determine the developmental stage to which that module was associated. In addition, we compared module variability for multiple (interspecific) vs. single species (intraspecific) analyses. For each case, all the modules that weighted positively on a spectrum were considered, and the total number of modules, average and standard deviation of eigenvalues were calculated per spectrum. We used a generalized model to test the differences between multiple and single species analyses for the calculated parameters.

## Results

### Prepared mixture application

**Figure 1:**
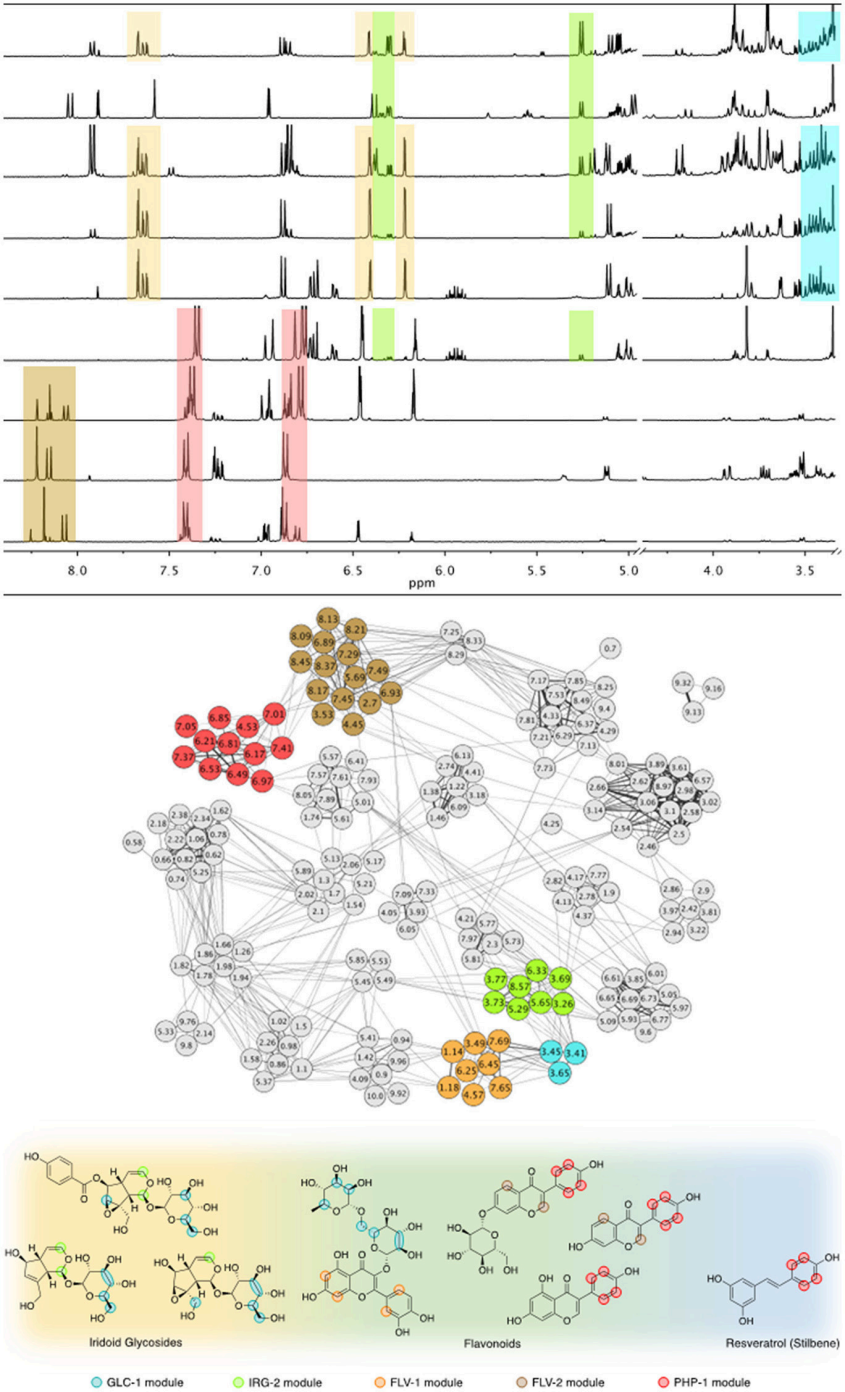
Overview of the network analysis, showing network construction and interpretation for the interclass mixtures set. Co-occurring ^1^H-NMR peaks are identified from the array of sample spectra (top) and attributed to a color-coded module. Here we highlight five of these modules that span through three classes of natural products. In the resulting network (center), the corresponding modules are colored to show complete node composition and module-to-module connectivity. The modules are properly named according to their compound class identity (bottom). FLV-2 module represents exclusively flavonoids, as it contains chemical shifts representative of specific molecular features of this class of compounds. It is interconnected to PHP-1, a phenylpropanoid module, due to the *p*-phenol moiety shared between flavonoids and the stilbene resveratrol. Another flavonoid module, FLV-1, is characteristic to the structural features of the compound rutin, and is connected to the module GLC-1, which represents glycosyl moieties. Since this is also a structural feature present in iridoid glycosides, GLC-1 is connected to the iridoid-specific module IRG-2.

In all three prepared mixture analyses (intraclass, interclass, and 4-component), the ^1^H-NMR network-based approach identified modules of co-occurring chemical shifts that were correlated with specific compounds or groups of chemical shifts that are characteristic of structural features shared by different compounds (Figure 1). Even with a broader range of compound combinations, the networks still produced coherent module-compound associations (Table 1). The Supplementary Information includes a table for each analysis (intraclass, interclass, and 4-component) with specific details on the proton resonances associated with each module identified, the compounds highly correlated with each module and the specific proton resonances indicated on the compounds (Tables S2–S4). Arbitrary color names were initially assigned to each module, so for the ease of discussion between analyses, we labeled the modules based on the consistent structural features identified across all three analyses (Tables S5–S6).

For intraclass mixtures, the technique was effective at distinguishing shared chemical features among relatively homogeneous samples (Figure S1, soft threshold β = 16). Each module was consistently correlated with compounds sharing structural features and often relating to compound class. Due to the high degree of compound co-occurrence in the intraclass analysis, some modules had a significant positive correlation with the concentration of compounds that were in the mixtures, but did not have peaks that were representative of that module. For example, since PBA (prenylated benzoic acid) was present in all the mixtures containing eugenol, it was not surprising that a module in the network was positively correlated with both compounds. The PHP-3 module was significantly correlated with PBA concentration, yet did not contain the representative peaks of PBA (Figure S1). This module had a higher positive correlation with eugenol and did include peaks specific to that compound. The analysis also identified chemical features shared across classes of compounds, such as the prenyl group of PBA and terpenoids in the STR-2 module.

Due to the increased mixture complexity in the interclass and the 4-component mixtures, the co-occurrence of compounds had a lesser influence on module affiliation. This complexity is a better reflection of naturally occurring mixtures and facilitated sensitivity tests for compound concentration and peak overlap found in such mixtures. These networks not only retained compound class-specificity of the modules, but they were also characterized by proton resonances particular to molecular features that were shared by compounds from distinct biosynthetic pathways. For example, in the interclass mixture analysis, the module PHP-1 (Table S3 and Figure 1) was correlated with flavonoids genistein and daidzein, and the stilbene resveratrol. This correlation was driven by chemical shifts from a shared aromatic ring derived from the phenylpropanoid pathway. Flavonoids and iridoids also shared a module due to their common glycosylated moieties (Table S3—module GLC-1—and Figure 1).

An important case was observed with amides and alkaloid modules, which were interconnected through specific proton resonances vicinal to the nitrogen atoms (Figure S2). In the interclass mixture analysis, the three amides and the alkaloid brucine were associated with module AMD-1, mostly due to proton resonances in the α- and β-position of nitrogen atoms. In the network, this module is strongly linked (the edges are due to related peaks) to two alkaloid-related modules, and together they are part of a cluster (or meta-module) of five modules that were indicative of nitrogen-containing compounds. The amides and alkaloids used in the analysis also share molecular features with other classes of compounds accounting for the connections with other modules. This example illustrates the utility of network analysis for identifying structural similarities in mixtures due to related chemical features. Another interesting and useful result demonstrates that the network analysis provided evidence of peaks originating from the interactions of compounds. The phenolic peaks in resveratrol are generally broad and undetectable, but the almost negligible resonances at δ 9.10 and δ 9.30 from the mixtures containing resveratrol, escin and oleanic were evident in module PHP-4. Phenolic peaks are sensitive to intermolecular interactions based on hydrogen bonding, as they reduce proton exchange and improve peak sharpness (Charisiadis et al., 2014). We verified experimentally that these peaks only appear in the presence of escin, suggesting that resveratrol in solution had hydrogen-bonding interactions with the glycosylated portion of that compound. This example demonstrates that the network analysis approach is highly sensitive to peak intensity and can identify features *only* present when specific combinations of compounds are present.

Given that each network generated from the prepared mixtures resulted in distinct module-peak-compound associations, we evaluated the efficacy of the approach for determining the presence of a known compound. This provided validation of the method consistency across mixtures with different compositions and complexities, and demonstrated that the analysis can be reproduced in other sampling designs, such as extracts from a diversity of field-collected plants or animals. However, precise validation, via various accuracy parameters can be challenging, given that not every peak in a spectrum is relevant in identifying a compound. For that reason, we opted to calculate accuracy as a simple ratio between peaks identified in the most correlated compound in a module and the total number of proton resonances expected from that compound (Table 1). The average overall accuracy was similar across all analyses (0.56 ± 0.05, 0.58 ± 0.05, and 0.52 ± 0.05 for intraclass, interclass, and 4 compound mixtures respectively) indicating that about 55% of the signals were captured in the most representative module of a compound. There were some modules that had a relatively low accuracy ratio. In many of these cases, the compounds had a large number of protons resonances (e.g., escin and digitoxin) and the protons from specific structural features were identified in the modules, but they did not represent the majority of all the protons present on the compound.

### Interspecific *Piper* application and bioactivity

**Figure 2:**
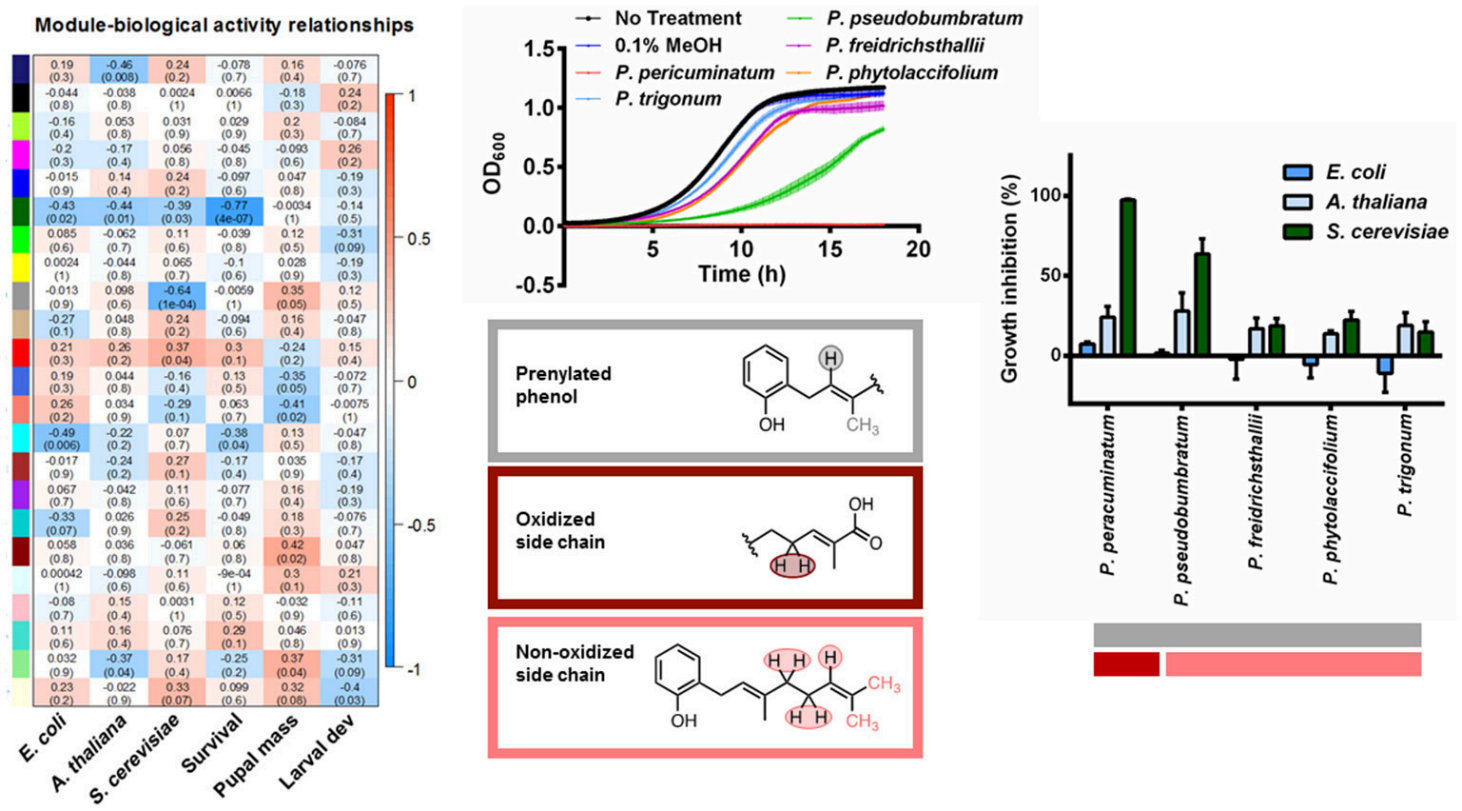
A correlation heatmap of module eigenvalue and bioactivity of *E. coli, A. thaliana, S. cerevisiae* (OD_600_ inset) and *S. exigua*. We identified compounds containing prenylated phenols across multiple *Piper* species with different side chains and different bioactivity.

For the networks of plant extracts, seeded by the laboratory generated mixtures, we set the soft threshold β at 11 and identified 23 modules. Unlike the networks based on artificial mixtures, the correlation matrices between modules and the compound concentrations of the prepared mixtures were more diffuse (Figure S3), with modules exhibiting positive loadings from more than one compound. As expected, due to extract complexity, the modules were characterized by shared compound moieties rather than capturing the majority of representative peaks of a specific compound. Nevertheless, network results were successfully combined with the bioassay data to aid in the identification of potential bioactive molecules. Most notably, we found that yeast growth was negatively correlated with the presence of peaks identified by the gray module (δ 1.74, 1.78, 1.82, 5.33). From the laboratory mixture networks, we determined that PBA was strongly correlated to this gray module, and more generally, these chemical shifts were representative of protons from a prenylated phenol. Based on the module eigenvalues, we identified five species that had these resonances (Figure 2, *P. peracuminatum, P. psuedobumbratum, P. friedrichsthalii, P. phytolaccifolium, P. trigonum*). The eigenvalues of other modules associated with these species revealed that the salmon module was positively associated with all species but one, *P. peracuminatum*, which was the source of the most active extract against yeast growth. In the prepared mixtures, the salmon module (δ 1.66, 1.70, 2.02, 2.06, 2.10, 2.14, and 5.13) was characterized by PBA and the terpenoids phytol and nerolidol (Figure S1), compounds whose spectra are strongly influenced by unsaturated aliphatic chains (prenyl groups). The spectra of the more toxic extracts of *P. peracuminatum* had an eigenvalue associated with the dark red module (δ 2.86, 4.25, and 4.29) which in the prepared mixtures network featured protons on the oxygenated moieties of digitoxin and pipleroxide. We hypothesize that these differences represent an analog compound of PBA that contains a more oxidized prenyl moiety, and this could be an influential factor in the increased antifungal activity of *P. peracuminatum* extracts. Furthermore, we identified small structural differences of related structures that may have a large effect on bioactivity. Additional research is necessary for isolation, structure determination, and bioactivity confirmation, nevertheless, this result exemplifies how the network approach outlined here can link spectral features to biological activity and, potentially, ecological interactions.

### Intraspecific *Piper* application and ontogeny

**Figure 3:**
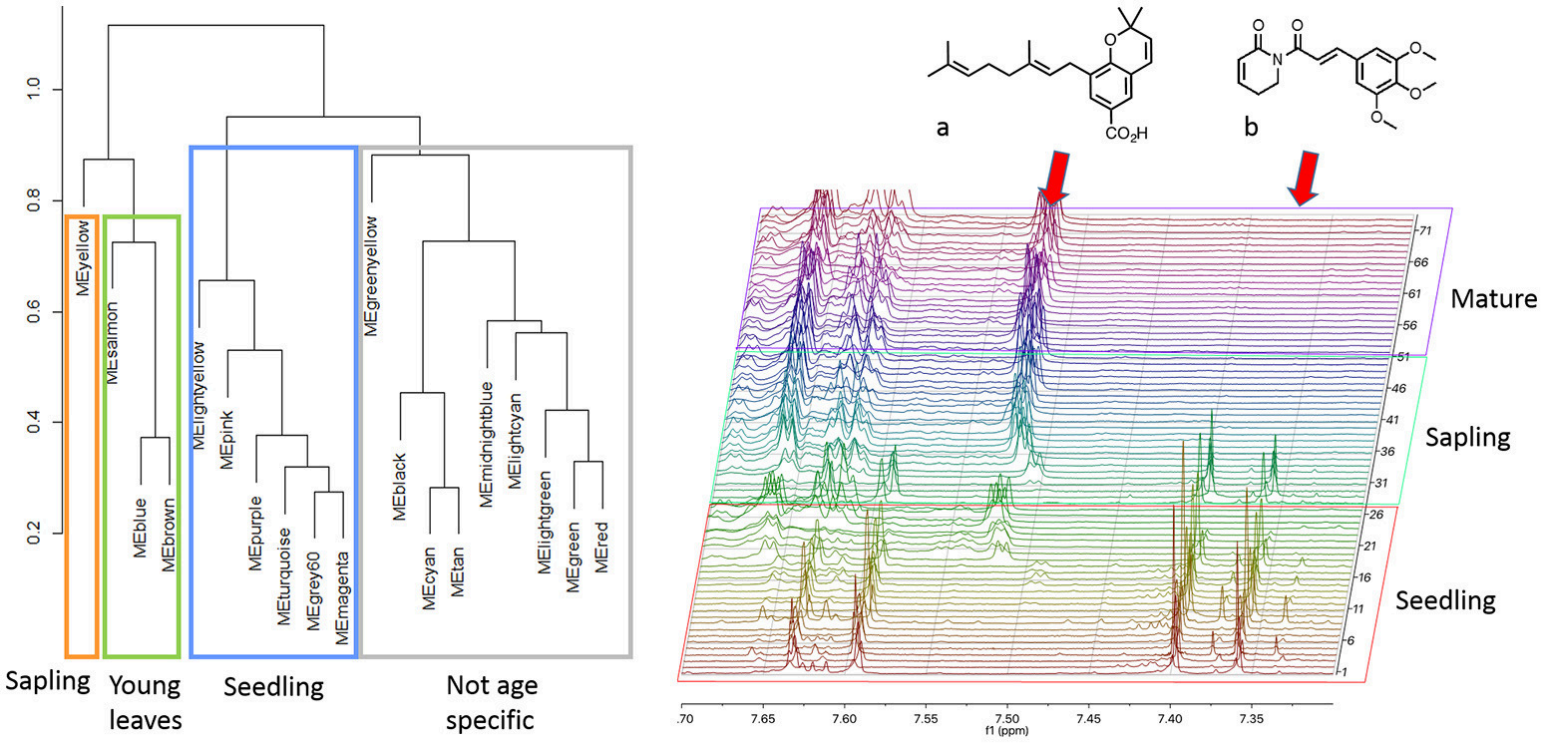
Intraspecific analysis of *Piper kelleyi* identified compounds present across life stages and compounds unique to seedlings and young leaves. A subset of the ^1^H-NMR spectra demonstrates the peaks associated with a chromene compound (a) and piplartine amide (b).

While the laboratory mixtures and interspecific *Piper* extracts exhibited a high degree of dissimilarity between spectra and yielded networks based on shared structural moieties, the *P. kelleyi* extracts were utilized to analyze similar spectra typically encountered in intraspecific comparisons. We identified 19 modules (soft threshold β = 6) in *P. kelleyi* that corresponded mostly to the representative protons of specific compounds. To determine the utility of the network for categorizing ontogeny, which is a very important factor generating plasticity in secondary metabolism (Koricheva, 2012) we utilized a hierarchical cluster analysis of the module eigenvalues, which clustered the modules into three main groups (Figure 3a). While some modules were not associated with a specific leaf age or plant stage, half of the modules identified were significantly associated with specific life stages (MANOVA, Wilks λ = 0.42 and 0.10 for leaf age and plant stage respectively *p* < 0.001). A cluster of six modules were specific to seedlings (Tukey's HSD *p* < 0.05), which according to the ^1^H-NMR spectra, are characterized by the amide piplartine and structurally similar amides in crude extracts. Piplartine was essentially absent in mature plants, where the peaks for a previously reported chromene (Jeffrey et al., 2014) are dominant (Figure 3b). The distinction between sapling and mature plants was not evident, as both contain peaks for chromene and its benzopyran dimer. Since these two compounds have a high degree of peak overlap, the modules related to these two stages were determined by a combination of the peaks for these two compounds. However, based on the resonances identified in the aromatic/alkene region of the spectrum, it is apparent that the sapling-related module are more influenced by peaks characteristic to the chromene, while the adult related modules were determined by the dimeric compound. This observation is consistent with the proposed biosynthetic origin of the dimeric chromane and photochemical studies of the relationship between the chromene and the dimeric chromane (Jeffrey et al., 2014)

### Interspecific vs. intraspecific module variation

Specifics of how modules were identified and weighted on all spectra differed between the multi vs. single species approaches (Table S7). In the analysis with multiple *Piper* species, spectra for each sample had more representative modules (10.45 ± 0.51, *t*-value = −4.66, *p* < 0.001) with lower average eigenvalues (0.082 ± 0.006, *t*-value = 3.36, *p* = 0.001) across samples. In comparison, each sample in the single species analysis had fewer represented modules (7.84 ± 0.43) with higher average eigenvalues (0.110 ± 0.005). There was no significant difference in the variation of the eigenvalues between the analyses (0.097 ± 0.006, 0.085 ± 0.005 for multiple and single species analysis respectively, *t*-value = −1.56, *p* = 0.12).

## Discussion

The methodology presented here demonstrates the potential of ^1^H-NMR networks to expose chemical markers of interest from complex mixtures. Through the use of prepared mixtures of secondary metabolites, we verified the reliability of the analysis for linking groups of ^1^H-NMR resonances to structurally similar compounds and identifying class-specific modules for metabolites. By varying the complexity of the prepared mixtures, we gained insight into compound correlations, such as relationships driven by secondary structural features (e.g., prenyl and glycosyl moieties in aromatic compounds), shared core features (Nitrogen-vicinal resonances in amides and alkaloids), and potentially from intramolecular interactions (resveratrol in the presence of escin). These results are based on a compound set that represents only part of the highly diverse structural space of plant secondary metabolites. We project that complementing the network with more complex and unique molecules found in complex extracts, as well as compounds originated from biosynthetic pathways other than those represented here, will enhance the power of this approach to recognize specific molecular features and facilitate structure elucidation of unknown compounds in other plant families. We also predict that this approach could be used in the investigation of non-model organisms besides plants, given the proper considerations regarding the general composition of the study system, and with the selection of representative compounds for the mixture training set.

The ability to identify shared structural features rather than absolute compound identities is important for the analysis of plant extracts, in which metabolite diversity and spectral complexity are more expressive. In the interspecific comparison of *Piper* species, we found higher module count per spectra, which was due to multiple shared structural features identified across species. The stability of modules within a species is an important attribute of this approach, making it more robust to phenotypic plasticity than for quantifying specific compounds, since individual compounds can vary substantially and may even drop out at certain ontogenetic stages. We found a strong positive correlation between yeast growth inhibition and the presence of specific chemical markers shared across different species. These markers appear to be related to a common structural feature present in related molecules: a prenyl group. The most active extract showed strong correlation to resonances that indicate a slightly modified version of the prenyl moiety, and the ongoing targeted isolation of the bioactive compound should confirm that hypothesis. Here, the network analysis and resulting eigenvalues connected chemical information (^1^H-NMR data) to biological activity across taxa, revealing important structure-activity relationships and facilitating the identification of compounds of interest. The chemistry of these species is largely unknown and the genus *Piper* is known to utilize divergent biosynthetic pathways between species, with closely related species producing different classes of compounds. Thus, this analysis also revealed potentially shared biosynthetic pathways that were previously unknown in these species, suggesting that the same method could be applied to connect secondary metabolic profiles to phylogenetic data, exposing specific relationships between compounds or specific compound features and their underlying genes. These results will require subsequent isolation, characterization and bioassays, nevertheless this example highlights the utility of the network based approach for identifying potential compounds of interest.

In the intraspecific study of *Piper kelleyi*, we combined the variable reduction resulting from network analysis and classic statistical methods to elucidate the effects of plant ontogeny on chemical composition. Given the lower variability in data for the *Piper* extracts, more information was extracted for specific compounds, resulting in fewer modules identified per sample. In the single species analysis the module eigenvalues were higher per sample due to higher sensitivity to compound concentrations. The modules revealed that piplartine was found in seedlings but was not present in other developmental stages. These results corroborate findings from a previous study on the changes in secondary metabolism during ontogeny in *P. gaudichaudianum* (Gaia et al., 2014). This species also produces a PBA in the seedling stage, which is postulated to be the precursor for the chromene in *P. kelleyi* (Jeffrey et al., 2014). The approach described here can be used to uncover broad patterns of change across ontogeny of plants, and those taxa that exhibit the greatest changes in eigenvalues or network structure warrant further chemical ecology investigation. For example, it is interesting to note the prevalence of amides in the seedlings, which are especially vulnerable to generalist herbivores, fungi, and other parasites. Amides, including piplartine, are produced by several species of *Piper*, where they function as antifungal and anti-herbivore defensive compounds (Navickiene et al., 2000; da Silva et al., 2002; Dyer et al., 2004; Marques et al., 2007, 2010). We hypothesize that these ontogenetic changes in defensive chemistry of *P. kelleyi* may reflect the changes in sources of mortality across the different life stages of a plant. As a seedling, plants may be more susceptible to fungal attack and generalist herbivory. Previous studies on *P. kelleyi* found that plants producing higher amounts of PBA and chromene had a lower diversity of specialist caterpillars, suggesting that these compounds play a defensive role against herbivory (Jeffrey et al., 2014; Tepe et al., 2014; Glassmire et al., 2016). Another study found that chromenes produced by species of *Encelia* (Asteraceae) were phototoxic to bacteria, yeast and insects (Proksch et al., 1983). The hypothesis of age-specific specialized chemical defenses is clarified by the biosynthetic evidence that piplartine and PBA originate in the PHP pathway, so metabolic flexibility is made possible by the existence of a common precursor, *p*-cinnamic acid. Network analysis on *P. kelleyi* successfully revealed an ontogenetic change in chemotype, however additional controlled studies should verify the gradual changes in metabolic profile during the development of *P. kelleyi* individual plants.

This method can be applied to any ^1^H-NMR dataset and is not limited to plant samples. There are several considerations for any system. First, the strength of the peak-module-compound associations will vary depending on the variation between samples. For example, in the interspecific analysis, modules identified shared structural features as opposed to complete compounds. In comparison, the modules in the single species analysis represented entire compounds or more complete fragments of compounds. Second, this method does not require seeding the analysis with known compounds, however it can be a useful tool for structure determination and de-replication. When including spectra of known compounds, it is helpful to choose compounds that would represent potential structural features based on previous knowledge of the samples. Since the analysis is based on co-variation among peaks across spectra, it is important to prepare the samples of known compounds with varying relative concentrations. And finally, it is possible that bioactivity associated with a module may be due to synergistic effects with compounds at concentrations not detected by the ^1^H-NMR and this analysis should be viewed as a starting point for further investigations.

It is relevant to point out a complementary approach, statistical total correlation spectroscopy (STOCSY), which is a method to identify co-varying peaks across sets of spectra ^1^H-NMR (Sands et al., 2011). Starting from a specific “driver” peak pre-defined by the analyst, the technique primarily identifies other peaks related to the same compound, and with multiple rounds of analysis it could also provide fine-tuned information about positive or negative compound associations. We support the view that important ^1^H-NMR peaks revealed through the proposed network approach could be seeded into STOCSY, from which we would further gain information to completely identify compounds of interest. This combination of techniques could also highlight unexpected metabolite associations, and therefore, motivate new hypothesis regarding the natural role of secondary metabolites in ecological systems.

## Conclusion

Plant secondary metabolite profiles vary within a species, across species, across habitats, and along gradients from local to global scales. However, there are not many established metrics that accurately categorize and quantitatively compare these mixtures, so it is tempting to focus on single compounds, broad-range colorimetric assays, or bioassays (Dyer et al., 2014, 2018). Here, we demonstrate that network analysis of ^1^H-NMR spectra can provide a heuristic summary of complex phytochemical mixtures. This approach can facilitate examination of the biological consequences of complete biosynthetic products, as opposed to focusing on effects of single compounds. It provides a method for identifying ecologically important chemical features that may be shared across compounds or active mixtures. From the perspective of natural products chemistry, this approach has the potential to facilitate filtering of extract arrays from multi-species field sampling, allowing one to focus on extracts that are characterized by the most promising network modules for subsequent targeted isolation and structure determination. For chemical ecology, it provides a tool to quantifying entire arrays of chemical defense within plant or animal tissues and using the parameters as predictors or response variables in statistical models. Module importance or overall network parameters can be examined in response to manipulations of resources or can be mapped onto phylogenies to address interesting questions about the origins of biodiversity (Ehrlich and Raven, 1964).

## Author contributions

LR, CJ, and LD designed the study. CO and AR prepared the samples and acquired the spectra. LR analyzed the data. CO validated resonances to compound associations. IW and FU-M performed and analyzed bioassays. CJ and CD interpreted ^1^H-NMR of plant extracts. The manuscript was written through contributions of all authors. All authors have given approval to the final version of the manuscript.

### Conflict of interest statement

The authors declare that the research was conducted in the absence of any commercial or financial relationships that could be construed as a potential conflict of interest.

## Supplementary material

NMR parameters, all module, resonance, and compounds associations for the different experiments and a heatmap of intraclass mixtures, N-based compounds in a network and a heatmap of *Piper* extract and prepared mixture analsyis and sample R code(PDF).
