# Supplementary material for: Shedding Light on Chemically Mediated Tri-Trophic Interactions: A 1H-NMR Network Approach to Identify Compound Structural Features and Associated Biological Activity
Source: Front Plant Sci. 2018 Aug 17;9:1155. doi: 10.3389/fpls.2018.01155 (PMC6107749; doi:10.3389/fpls.2018.01155)
Supplement: Supplementary file 1 [file Table_1.DOCX]

Table S1. ^1^H-NMR experiment parameters used in the analysis of the prepared mixtures and crude plant extracts.

| **Experiment Parameter** | **Value/Description** |
| --- | --- |
| Acquisition |  |
| Pulse program | s2pul |
| Sweep widths (sw) | 6410.3 |
| Acquisition time (at) | 2.556 s |
| Number of points (np) | 32768 |
| Delay time (d1) | 1.000 s |
| Pulse Angle | 45° |
| Transmitter |  |
| Frequency (sfrq) | 399.773 MHz |
| Offset (tof) | 399.8 MHz |
| RF Pulse power level (tpwr) | 60 dB |
| Pulse width (pw) | 8.800 μs |
| Additional |  |
| Homospoil time (hst) | 0.008 ms |
| pw90 | 17.600 s |
| Alfa delay before acquisition (alfa) | 10.000 μs |
| Presaturation | Not used |
