# Supplementary material for: Shedding Light on Chemically Mediated Tri-Trophic Interactions: A 1H-NMR Network Approach to Identify Compound Structural Features and Associated Biological Activity
Source: Front Plant Sci. 2018 Aug 17;9:1155. doi: 10.3389/fpls.2018.01155 (PMC6107749; doi:10.3389/fpls.2018.01155)
Supplement: Supplementary file 2 [file Table_2.DOCX]

Table S2. Module identity, chemical shifts and compound correlations obtained from the network analysis of intraclass mixtures. Modules are named accordingly to the color code generated in the analysis, and the unified code (in parenthesis) that best describes the highlighted structural features. The representative compounds for each module are shown with their respective correlation value. The colored circles indicate proton resonances depicted by the module, whose values in ppm are displayed under the module name. Unfilled circles identified resonances within 0.1 ppm of an identified bin. Chemical shift values with no correspondence to the molecules of the module are indicated in black.

| **MODULE (δ)** | **COMPOUNDS (Pearson's correlation)** |
| --- | --- |
| **BROWN (PHP-1)**  **6.17 6.21 6.49 6.53 6.81 6.97 7.37 9.05 9.09 9.13 9.20 9.28** 9.48 | **Resveratrol (0.93)** |
| **GREY 60 (PHP-2)**  **1.74 2.14 2.18 5.33 9.76 9.80** | **Eugenol (0.48)** |
|  | **PBA (0.97)** |
|  | **Resveratrol (0.46)** |
| **CYAN (PHP-3)**  **5.93 5.97 6.61 6.65 6.69 6.73 6.77** | **Eugenol (0.97)** |
|  | **PBA (0.68)** |
|  | **Resveratrol (0.68)** |
| **PINK (TPN-1)**  **0.62 0.66** 0.70 **0.74 0.78 0.82 1.06 1.62 2.22 2.34 5.25** | **Carene (0.92)** |
|  | **Nerolidol (0.58)** |
|  | **Phytol (0.51)** |
| **SALMON (TPN-2)**  **0.58 1.30 1.54 1.70 2.02 2.06 2.10 5.89** | **Carene (0.61)** |
|  | **Nerolidol (0.94)** |
|  | **Phytol (0.61)** |
|  | **Phytenal** |
| **LIGHT CYAN**  **(TPN-3)**  **0.90 0.94 1.34 1.42 4.09 9.96 10.0** | **Carene (0.57)** |
|  | **Nerolidol (0.57)** |
|  | **Phytenal** |
|  | **Phytol (0.95)** |
| **ROYAL BLUE**  **(STR-1)**  **5.49 5.53** 7.89 | **Escin (0.84)** |
| **LIGHT YELLOW**  **(STR-2)**  **1.66 1.78 1.82** | **Diosgenin (0.63)** |
|  | **Escin (0.54)** |
|  | **Oleanic acid (0.56)** |
|  | **PBA (0.52)** |
| **TURQUOISE**  **(STR-3)**  **0.86 0.98 1.02 1.10 1.18 1.26 1.50 1.58 1.86 1.94 1.98 2.26 5.37 5.41** 5.45 7.81 | **Diosgenin (0.81)** |
|  | **Escin (0.81)** |
|  | ****  **Oleanic acid (0.77)** |
|  |  |
| **LIGHT GREEN (ALK-1)**  **1.22 1.38 1.46 6.09 6.13** | **Boldine (0.52)** |
|  | **Brucine (0.52)** |
|  | **Crotaline (0.96)** |
| **YELLOW (ALK-2)**  **2.46 2.50 2.58 2.98 3.02 3.06 3.10 3.14 3.18 3.61 3.89 6.57 8.01** | **Boldine (0.97)** |
|  | **Brucine (0.66)** |
|  | **Crotaline (0.64)** |
| **GREEN YELLOW**  **(ALK-3)**  **2.74 2.78 2.82 4.13 4.17 4.33 4.37 7.77** | **Boldine (0.55)** |
|  | **Brucine (0.92)** |
|  | **Crotaline (0.53)** |
| **PURPLE (AMD-1)**  **2.42 2.86 2.90 2.94 3.22 3.81 4.25 4.29 7.05** | **Alkene amide (0.86)** |
|  | **Piplartine (0.67)** |
|  | **Pipleroxide (0.90)** |
| **MAGENTA (AMD-2)**  **2.54 3.93 4.01 4.05 6.05 7.09 7.13 7.33 7.61 7.65** | **Alkene amide (0.52)** |
|  | **Piplartine (0.99)** |
|  | **Pipleroxide (0.53)** |
| **GREEN (FLV-1)**  **3.41 3.45 4.49 3.57 4.49 4.53 4.57 5.21 6.25 6.45 7.69 7.73** 9.64 | **Daidzein (0.51)** |
|  | **Daidzin (0.49)** |
|  | **Rutin (0.93)** |
| **MIDGNIGHT BLUE**  **(IRG-1)**  **3.77** 5.61 **6.41** 7.53 7.57  **7.93 7.97** | **Aucubin (0.51)** |
|  | **Catalpol (0.54)** |
|  | **Catapolside (0.99)** |
| **TAN (IRG-2)**  **3.69 3.73 5.29** 5.65 **6.33** 8.57 10.68 10.96 11.0 11.28 11.84 11.88 | **Aucubin (0.50)** |
|  | **Catalpol (0.97)** |
|  | **Catapolside (0.66)** |
| **BLACK (IRG-3)**  **2.30 3.26 3.65 4.21 5.77 5.81 5.85 6.33**  **6.37** | **Aucubin (0.95)** |
|  | **Catalpol (0.75)** |
|  | **Catapolside (0.72)** |
| **BLUE (FLV-2)**  **2.70 4.45 6.29 6.93** 7.29 **7.45 7.49 8.09 8.21** 8.29 8.33 8.37 8.45 | **Daidzein (0.97)** |
|  | **Rutin (0.43)** |
| **RED (FLV-3)**  2.62 2.66 **3.53** 4.41 **7.21 7.25 7.41 8.05 8.13 8.17 8.25** 8.49 8.97 | **Daidzein (0.43)** |
|  | **Daidzin (0.96)** |
| **DARK RED**  **(IRG-4)**  **5.01 5.05 5.09** | **Aucubin (0.5)** |
|  | **Catalpol (0.47)** |
|  | **Catapolside (0.47)** |
|  | **Eugenol (0.72)** |
