# Supplementary material for: Shedding Light on Chemically Mediated Tri-Trophic Interactions: A 1H-NMR Network Approach to Identify Compound Structural Features and Associated Biological Activity
Source: Front Plant Sci. 2018 Aug 17;9:1155. doi: 10.3389/fpls.2018.01155 (PMC6107749; doi:10.3389/fpls.2018.01155)
Supplement: Supplementary file 3 [file Table_3.DOCX]

Table S3. Module identity, chemical shifts and compound correlations obtained from the network analysis of interclass mixtures. Modules are named accordingly to the color code generated in the analysis, and the unified code (in parenthesis) that best describes the highlighted structural features. The representative compounds for each module are shown with their respective correlation value. The colored circles indicate proton resonances depicted by the module, whose values in ppm are displayed under the module name. Unfilled circles identified resonances within 0.1 ppm of an identified bin. Chemical shift values with no correspondence to the molecules of the module are indicated in black.

| **MODULE (δ)** | **COMPOUNDS (Pearson's correlation)** |
| --- | --- |
| **ROYAL BLUE**  **(PHP-2)**  **2.14 5.33 9.76 9.80** | **PBA (0.95)** |
| **TAN (TPN-3)**  **0.90 0.94 1.42 4.09 5.41 9.92 9.96 10.0** | **Carene (0.19)** |
|  | **Escin (0.26)** |
|  | **Nerolidol (0.18)** |
|  | **Phytol (0.89)** |
|  | **Phytenal** |
| **PINK (TPN-2)**  **1.30 1.54 1.70 2.02 2.06 2.10 5.13 5.17 5.21 5.89** | **Carene (0.22)** |
|  | **Phytol (0.24)** |
|  | **Phytenal** |
|  | **Nerolidol (0.9)** |
| **YELLOW (TPN-1)**  **0.58 0.62 0.66 0.74 0.78 0.82 1.06 1.62 2.18 2.22 2.34 2.38 5.25** | **Carene (0.94)** |
|  | **Nerolidol (0.3)** |
|  | **Phytol (0.19)** |
| **MAGENTA**  **(STR-3)**  **0.70 0.86 0.98 1.02 1.10 1.50 1.58 2.26 5.37** | **Diosgenin (0.63)** |
|  | **Escin (0.35)** |
|  | **Oleanic Acid (0.5)** |
|  | **Sitosterol (0.35)** |
|  | **Stigmasterol (0.21)** |
| **MIDNIGHT BLUE**  **(STR-2)**  **1.26 1.66 1.78 1.82 1.86 1.94 1.98** | **Carene (0.33)** |
|  | **Digitoxin (0.33)** |
|  | **Diosgenin (0.5)** |
|  | **Escin (0.52)** |
|  | **Nerolidol (0.15)** |
|  | **Oleanic Acid (0.32)** |
|  | **PBA (0.22)** |
| **LIGHT YELLOW**  **(STR-1)**  **5.45 5.49 5.53 5.85** | **Escin (0.91)** |
|  | **Quillaja (0.33)** |
|  |  |
| **PURPLE (ALK-1)**  **1.22 1.38 1.46 2.74 3.18 4.41 6.09 6.13** | **Aucubin (0.16)** |
|  | **Crotaline (0.98)** |
| **TURQUOISE**  **(ALK-2)**  **2.46 2.50 2.54 2.58 2.62 2.66 2.98 3.02 3.06 3.10 3.14 3.61 3.89 6.57 8.01** 8.97 | **Boldine (0.96)** |
|  | **Brucine (0.29)** |
|  | **Crotaline (0.17)** |
| **CYAN (ALK-3)**  **1.90 2.78 2.82 4.13 4.17 4.37 7.77** | **Aucubin (0.25)** |
|  | **Brucine (0.9)** |
|  | **Caffeine (0.2)** |
| **BLACK (FRC-1)**  **1.74 5.01 5.57 5.61 6.41 7.57 7.61 7.89 7.93 8.05** | **Aucubin (0.15)** |
|  | **Imperatorin (0.91)** |
|  | **Xanthotoxin (0.15)** |
| **SALMON (FLV-1)**  **1.14 1.18 3.49 4.57 6.25 6.45 7.65 7.69** | **Daidzein (0.16)** |
|  | **Rutin (0.92)** |
| **GREY 60**  **(IRG-3)**  2.30 **4.21 5.73 5.77 5.81 7.97** | **Aucubin (0.77)** |
|  | **Catapolside (0.43)** |
| **GREEN YELLOW**  **(IRG-2)**  **3.26 3.69 3.73 3.77 5.29** 5.65 **6.33** 8.57 | **Aucubin (0.23)** |
|  | **Catalpol (0.88)** |
|  | **Catapolside (0.34)** |
| **BLUE (FRC-2)**  **4.25 4.29 4.33 6.29 6.37 7.13 7.17 7.21 7.53 7.73 7.81 7.85 8.25** 8.49 9.40 | **Bergapten (0.96)** |
|  | **Xanthotoxin (0.77)** |
| **BROWN**  **(FLV-2)**  2.70 **3.53** 4.45 5.69 **6.89 6.93 7.29 7.45 7.49 8.09 8.13 8.17 8.21** 8.37 8.45 | **Daidzein (0.8)** |
|  | **Daidzin (0.55)** |
| **LIGHT CYAN**  **(AMD-1)**  **2.42 2.86 2.90 2.94 3.22 3.81 3.97** | **Alkene Amide (0.77)** |
|  | **Brucine (0.19)** |
|  | **Piplartine (0.26)** |
|  | **Pipleroxide (0.53)** |
| **LIGHT GREEN (AMD-2)**  **3.93 4.05 6.05 7.09 7.33** | **Alkene Amide (0.18)** |
|  | **Digitoxin (0.23)** |
|  | **Piplartine (0.9)** |
|  | **Pipleroxide (0.38)** |
|  | **Quillaja Saponin (0.19)**  **** |
|  | **Stigmasterol (0.16)** |
| **GREEN (PHP-3)**  **3.85 5.05 5.09 5.93 5.97 6.01 6.61 6.65 6.69 6.73 6.77 9.60** | **Eugenol (0.91)** |
|  | **Resveratrol (0.25)** |
| **RED (PHP-1)**  4.53 **6.17 6.21 6.49 6.53 6.81 6.85 6.97 7.01** 7.05 **7.37 7.41** | **Daidzein (0.18)** |
|  | **Genistein (0.23)** |
|  | **Resveratrol (0.93)** |
| **DARK RED (PHP-4)**  **9.13 9.16 9.32** | **Oleanic Acid (0.17)** |
|  | **Resveratrol (0.34)** |
| **DARK TURQUOISE**  **(GLC-1)**  **3.41 3.45 3.65** | **Aucubin (0.31)** |
|  | **Catapolside (0.4)** |
|  | **Catalpol (0.24)** |
|  | **Rutin (0.6)** |
| **DARK GREEN**  **(FLV-3)**  **7.25 8.29** 8.33 | **Bergapten (0.96)** |
|  | **Daidzein (0.39)** |
|  | **Daidzin (0.55)** |
|  | **Xanthotoxin (0.4)** |
