# Supplementary material for: Shedding Light on Chemically Mediated Tri-Trophic Interactions: A 1H-NMR Network Approach to Identify Compound Structural Features and Associated Biological Activity
Source: Front Plant Sci. 2018 Aug 17;9:1155. doi: 10.3389/fpls.2018.01155 (PMC6107749; doi:10.3389/fpls.2018.01155)
Supplement: Supplementary file 4 [file Table_4.DOCX]

Table S4. Module identity, chemical shifts and compound correlations obtained from the network analysis of complex mixtures. Modules are named accordingly to the color code generated in the analysis, and the unified code (in parenthesis) that best describes the highlighted structural features. The representative compounds for each module are shown with their respective correlation value. The colored circles indicate proton resonances depicted by the module, whose values in ppm are displayed under the module name. Unfilled circles identified resonances within 0.1 ppm of an identified bin. Chemical shift values with no correspondence to the molecules of the module are indicated in black.

| **MODULE (δ)** | **COMPOUNDS (Pearson's correlation)** |
| --- | --- |
| **GREEN**  **(TPN-3) 0.90 0.94 0.98 1.10 1.22 1.42 1.46 1.50 4.09 5.37 5.41 9.96** | **Escin (0.31)** |
|  | **Phytol (0.76)** |
|  | **Phytenal (N/A)** |
|  | **Sitosterol (0.25)**  **** |
| **PURPLE (TPN-2)**  **1.54 1.58 1.66 1.70 2.02 2.06 2.10 2.14 5.89** | **Carene (0.23)** |
|  | **Phytol (0.32)** |
|  | **Phytenal** |
|  | **Nerolidol (0.93)** |
| **PINK (TPN-1)**  **0.62 0.66 0.78 0.82 1.06 1.62 2.34 2.38 5.25** | **Carene (0.95)** |
|  | **Nerolidol (0.25)** |
| **BLACK (STR-1)**  **1.94 1.98 4.45 5.45 5.49 5.53 5.77 5.81 5.85 6.13** | **Escin (0.88)** |
|  | **Quillaja Saponin (0.55)** |
| **MIDNIGHT BLUE (STR-2)**  **1.26 1.74 1.78 1.82 1.86 2.22** | **Carene (0.47)** |
|  | **Digitoxin (0.77)** |
|  | **Escin (0.28)** |
| **BROWN (ALK-2)**  **2.50 2.58 2.62 2.66 2.70 3.02 3.06 3.10 3.14 3.61 3.89 6.57** | **Boldine (0.95)** |
|  | **Catalpol (0.25)** |
| **TAN (ALK-3)**  **1.90 2.74 2.78 2.82 4.17 4.37 7.77** | **Brucine (0.97)** |
|  | **Caffeine (0.43)** |
| **RED (FLV-1)**  **1.14 1.18 3.41 3.45 3.49 3.65 4.53 4.57 6.45 7.69** | **Daidzein (0.59)** |
|  | **Daidzin (0.5)** |
|  | **Rutin (0.89)** |
| **YELLOW (IRG-1)**  **3.26 3.69 3.73 3.77 4.21 5.29** 5.57 5.61 5.65 **6.33 7.93** 8.57 | **Catalpol (0.8)** |
|  | **Catapolside (0.83)** |
| **TURQUOISE**  **(FRC-2)**  **4.29 4.33 6.29 6.41 7.17 7.21 7.25 7.53 7.81 7.85 8.01 8.25** 8.29 8.33 | **Bergapten (0.98)** |
|  | **Xanthotoxin (0.84)** |
| **LIGHT YELLOW (FLV-3)**  **7.29 8.17 8.21** | **Daidzin (0.73)** |
|  | **Rutin (0.49)** |
| **GREY 60 (AMD-1)**  **2.42 2.46 2.94 3.97 7.05** | **Alkene Amide (0.8)** |
|  | **Boldine (0.22)** |
|  | **Pipleroxide (0.22)** |
| **GREEN YELLOW**  **(AMD-2)**  **2.54 3.93 6.05 6.93 7.09 7.33 7.65** | **Piplartine (0.82)** |
|  | **Pipleroxide (0.4)** |
| **BLUE (PHP-3)**  **3.85 5.01 5.05 5.09 5.93 5.97 6.01 6.61 6.65 6.69 6.73 6.77** | **Eugenol (0.89)** |
|  | **Resveratrol (0.28)** |
| **CYAN**  **(FLV-2)**  **6.25 6.37 7.41 7.45 8.09 8.13** | **Genistein (0.88)** |
|  | **Resveratrol (0.35)** |
| **MAGENTA (PHP-1)**  **6.17 6.21 6.49 6.53 6.81 6.85 6.97 7.01 7.37** | **Genistein (0.35)** |
|  | **Resveratrol (0.91)** |
| **DARK RED (TPN-2)**  **5.13 5.17 5.21** | **Catalpol (0.23)** |
|  | **Catapolside (0.32)** |
|  | **Daidzin (0.23)** |
|  | **Nerolidol (0.76)** |
|  | **Rutin (0.26)** |
| **LIGHT CYAN**  **(FRC-2)**  **0.70 7.13 7.49 7.73 7.97** 9.40 | **Bergapten (0.47)** |
|  | **Sitosterol (0.26)** |
|  | **Xanthotoxin (0.38)** |
| **LIGHT GREEN (AMD-1)**  **2.86 2.90 3.18 3.22 3.81** | **Alkene Amide (0.53)** |
|  | **Piplartine (0.26)** |
|  | **Pipleroxide (0.69)** |
| **ROYAL BLUE**  **(STR-1)**  **4.49 9.48** 9.56 | **Escin (0.52)** |
|  | **Quillaja Saponin (0.88)**  **** |
| **SALMON (STR-3)**  0.58 **0.74 0.86 1.02 2.26 2.30 5.33** | **Sitosterol (0.76)** |
|  | ****  **Stigmasterol (0.42)** |
