# Supplementary material for: Shedding Light on Chemically Mediated Tri-Trophic Interactions: A 1H-NMR Network Approach to Identify Compound Structural Features and Associated Biological Activity
Source: Front Plant Sci. 2018 Aug 17;9:1155. doi: 10.3389/fpls.2018.01155 (PMC6107749; doi:10.3389/fpls.2018.01155)
Supplement: Supplementary file 5 [file Table_5.DOCX]

Table S5. Chemical shifts and their correspondence to the unified structural code for the three analysis. Modules are color coded accordingly to the main class of metabolites they represent, indicated by the boxes in the right column.

| ppm | Intraclass | Interclass | Complex |  |  |  |  |  |
| --- | --- | --- | --- | --- | --- | --- | --- | --- |
| 0.54 | - | - | - |  |  |  |  |  |
| 0.58 | TPN-2 | TPN-1 | STR-3 |  | MEVALONATE PATHWAY | **TPN-** TERPENOIDS | | |
| 0.62 | TPN-1 | TPN-1 | TPN-1 |  |  | **STR-** PHYTOSTEROLS/ SAPONINS | | |
| 0.66 | TPN-1 | TPN-1 | TPN-1 |  |  | **IRG** - IRIDOID GLYCOSIDES | | |
| 0.70 | TPN-1 | STR-3 | FRC-2 |  |  |  |  |  |
| 0.74 | TPN-1 | TPN-1 | STR-3 |  | NITROGEN BASED | **ALK -** ALKALOIDS | | |
| 0.78 | TPN-1 | TPN-1 | TPN-1 |  |  | **AMD -** AMIDES | | |
| 0.82 | TPN-1 | TPN-1 | TPN-1 |  |  |  |  |  |
| 0.86 | STR-3 | STR-3 | STR-3 |  | SHIKIMATE PATHWAY | **FLV-** FLAVONOIDS | | |
| 0.90 | TPN-3 | TPN-3 | TPN-3 |  |  | **PHP-** PHENYLPROPANOIDS | | |
| 0.94 | TPN-3 | TPN-3 | TPN-3 |  |  | **FRC­-** FURANOCOUMARINS | | |
| 0.98 | STR-3 | STR-3 | TPN-3 |  |  |  |  |  |
| 1.02 | STR-3 | STR-3 | STR-3 |  | **GLU -** GLUCOSE MOIETY | | | |
| 1.06 | TPN-1 | TPN-1 | TPN-1 |  |  |  |  |  |
| 1.10 | STR-3 | STR-3 | TPN-3 |  |  |  |  |  |
| 1.14 | - | FLV-1 | FLV-1 |  |  |  |  |  |
| 1.18 | STR-3 | FLV-1 | FLV-1 |  |  |  |  |  |
| 1.22 | ALK-1 | ALK-1 | TPN-3 |  |  |  |  |  |
| 1.26 | STR-3 | STR-2 | STR-2 |  |  |  |  |  |
| 1.30 | TPN-2 | TPN-2 | - |  |  |  |  |  |
| 1.34 | TPN-3 | - | - |  |  |  |  |  |
| 1.38 | ALK-1 | ALK-1 | - |  |  |  |  |  |
| 1.42 | TPN-3 | TPN-3 | TPN-3 |  |  |  |  |  |
| 1.46 | ALK-1 | ALK-1 | TPN-3 |  |  |  |  |  |
| 1.50 | STR-3 | STR-3 | TPN-3 |  |  |  |  |  |
| 1.54 | TPN-2 | TPN-2 | TPN-2 |  |  |  |  |  |
| 1.58 | STR-3 | STR-3 | TPN-2 |  |  |  |  |  |
| 1.62 | TPN-1 | TPN-1 | TPN-1 |  |  |  |  |  |
| 1.66 | STR-2 | STR-2 | TPN-2 |  |  |  |  |  |
| 1.70 | TPN-2 | TPN-2 | TPN-2 |  |  |  |  |  |
| 1.74 | PHP-2 | FRC-1 | STR-2 |  |  |  |  |  |
| 1.78 | STR-2 | STR-2 | STR-2 |  |  |  |  |  |
| 1.82 | STR-2 | STR-2 | STR-2 |  |  |  |  |  |
| 1.86 | STR-3 | STR-2 | STR-2 |  |  |  |  |  |
| 1.90 | - | ALK-3 | ALK-3 |  |  |  |  |  |
| 1.94 | STR-3 | STR-2 | STR-1 |  |  |  |  |  |
| 1.98 | STR-3 | STR-2 | STR-1 |  |  |  |  |  |
| 2.02 | TPN-2 | TPN-2 | TPN-2 |  |  |  |  |  |
| 2.06 | TPN-2 | TPN-2 | TPN-2 |  |  |  |  |  |
| 2.10 | TPN-2 | TPN-2 | TPN-2 |  |  |  |  |  |
| 2.14 | PHP-2 | PHP-2 | TPN-2 |  |  |  |  |  |
| 2.18 | PHP-2 | TPN-1 | - |  |  |  |  |  |
| 2.22 | TPN-1 | TPN-1 | STR-2 |  |  |  |  |  |
| 2.26 | STR-3 | STR-3 | STR-3 |  |  |  |  |  |
| 2.30 | IRG-3 | IRG-3 | STR-3 |  |  |  |  |  |
| 2.34 | TPN-1 | TPN-1 | TPN-1 |  |  |  |  |  |
| 2.38 | - | TPN-1 | TPN-1 |  |  |  |  |  |
| 2.42 | AMD-1 | AMD-1 | AMD-1 |  |  |  |  |  |
| 2.46 | ALK-2 | ALK-2 | AMD-1 |  |  |  |  |  |
| 2.50 | ALK-2 | ALK-2 | ALK-2 |  |  |  |  |  |
| 2.54 | AMD-2 | ALK-2 | AMD-2 |  |  |  |  |  |
| 2.58 | ALK-2 | ALK-2 | ALK-2 |  |  |  |  |  |
| 2.62 | FLV-3 | ALK-2 | ALK-2 |  |  |  |  |  |
| 2.66 | FLV-3 | ALK-2 | ALK-2 |  |  |  |  |  |
| 2.70 | FLV-2 | FLV-2 | ALK-2 |  |  |  |  |  |
| 2.74 | ALK-3 | ALK-1 | ALK-3 |  |  |  |  |  |
| 2.78 | ALK-3 | ALK-3 | ALK-3 |  |  |  |  |  |
| 2.82 | ALK-3 | ALK-3 | ALK-3 |  |  |  |  |  |
| 2.86 | AMD-1 | AMD-1 | AMD-1 |  |  |  |  |  |
| 2.90 | AMD-1 | AMD-1 | AMD-1 |  |  |  |  |  |
| 2.94 | AMD-1 | AMD-1 | AMD-1 |  |  |  |  |  |
| 2.98 | ALK-2 | ALK-2 | - |  |  |  |  |  |
| 3.02 | ALK-2 | ALK-2 | ALK-2 |  |  |  |  |  |
| 3.06 | ALK-2 | ALK-2 | ALK-2 |  |  |  |  |  |
| 3.10 | ALK-2 | ALK-2 | ALK-2 |  |  |  |  |  |
| 3.14 | ALK-2 | ALK-2 | ALK-2 |  |  |  |  |  |
| 3.18 | ALK-2 | ALK-1 | AMD-1 |  |  |  |  |  |
| 3.22 | AMD-1 | AMD-1 | AMD-1 |  |  |  |  |  |
| 3.26 | IRG-3 | IRG-2 | IRG-1 |  |  |  |  |  |
| 3.41 | FLV-1 | GLC-1 | FLV-1 |  |  |  |  |  |
| 3.45 | FLV-1 | GLC-1 | FLV-1 |  |  |  |  |  |
| 3.49 | FLV-1 | FLV-1 | FLV-1 |  |  |  |  |  |
| 3.53 | FLV-3 | FLV-2 | - |  |  |  |  |  |
| 3.57 | FLV-1 | - | - |  |  |  |  |  |
| 3.61 | ALK-2 | ALK-2 | ALK-2 |  |  |  |  |  |
| 3.65 | IRG-3 | GLC-1 | FLV-1 |  |  |  |  |  |
| 3.69 | IRG-2 | IRG-2 | IRG-1 |  |  |  |  |  |
| 3.73 | IRG-2 | IRG-2 | IRG-1 |  |  |  |  |  |
| 3.77 | IRG-1 | IRG-2 | IRG-1 |  |  |  |  |  |
| 3.81 | AMD-1 | AMD-1 | AMD-1 |  |  |  |  |  |
| 3.85 | - | PHP-3 | PHP-3 |  |  |  |  |  |
| 3.89 | ALK-2 | ALK-2 | ALK-2 |  |  |  |  |  |
| 3.93 | AMD-2 | AMD-2 | AMD-2 |  |  |  |  |  |
| 3.97 | - | AMD-1 | AMD-1 |  |  |  |  |  |
| 4.01 | AMD-2 | - | - |  |  |  |  |  |
| 4.05 | AMD-2 | AMD-2 | - |  |  |  |  |  |
| 4.09 | TPN-3 | TPN-3 | TPN-3 |  |  |  |  |  |
| 4.13 | ALK-3 | ALK-3 | - |  |  |  |  |  |
| 4.17 | ALK-3 | ALK-3 | ALK-3 |  |  |  |  |  |
| 4.21 | IRG-3 | IRG-1 | IRG-1 |  |  |  |  |  |
| 4.25 | AMD-1 | FRC-2 | - |  |  |  |  |  |
| 4.29 | AMD-1 | FRC-2 | FRC-2 |  |  |  |  |  |
| 4.33 | ALK-3 | FRC-2 | FRC-2 |  |  |  |  |  |
| 4.37 | ALK-3 | ALK-3 | ALK-3 |  |  |  |  |  |
| 4.41 | FLV-3 | ALK-1 | - |  |  |  |  |  |
| 4.45 | FLV-2 | FLV-2 | STR-1 |  |  |  |  |  |
| 4.49 | FLV-1 | - | STR-1 |  |  |  |  |  |
| 4.53 | FLV-1 | PHP-1 | FLV-1 |  |  |  |  |  |
| 4.57 | FLV-1 | FLV-1 | FLV-1 |  |  |  |  |  |
| 5.01 | IRG-4 | FRC-1 | PHP-3 |  |  |  |  |  |
| 5.05 | IRG-4 | PHP-3 | PHP-3 |  |  |  |  |  |
| 5.09 | IRG-4 | PHP-3 | PHP-3 |  |  |  |  |  |
| 5.13 | - | TPN-2 | TPN-2 |  |  |  |  |  |
| 5.17 | - | TPN-2 | TPN-2 |  |  |  |  |  |
| 5.21 | FLV-1 | TPN-2 | TPN-2 |  |  |  |  |  |
| 5.25 | TPN-1 | TPN-1 | TPN-1 |  |  |  |  |  |
| 5.29 | IRG-2 | IRG-2 | IRG-1 |  |  |  |  |  |
| 5.33 | PHP-2 | PHP-2 | STR-3 |  |  |  |  |  |
| 5.37 | STR-3 | STR-3 | TPN-3 |  |  |  |  |  |
| 5.41 | STR-3 | TPN-3 | TPN-3 |  |  |  |  |  |
| 5.45 | STR-3 | STR-1 | STR-1 |  |  |  |  |  |
| 5.49 | STR-1 | STR-1 | STR-1 |  |  |  |  |  |
| 5.53 | STR-1 | STR-1 | STR-1 |  |  |  |  |  |
| 5.57 | - | FRC-1 | IRG-1 |  |  |  |  |  |
| 5.61 | IRG-1 | FRC-1 | IRG-1 |  |  |  |  |  |
| 5.65 | IRG-2 | IRG-2 | IRG-1 |  |  |  |  |  |
| 5.69 | - | FLV-2 | - |  |  |  |  |  |
| 5.73 | - | IRG-3 | - |  |  |  |  |  |
| 5.77 | IRG-3 | IRG-3 | STR-1 |  |  |  |  |  |
| 5.81 | IRG-3 | IRG-3 | STR-1 |  |  |  |  |  |
| 5.85 | IRG-3 | STR-1 | STR-1 |  |  |  |  |  |
| 5.89 | TPN-2 | TPN-2 | TPN-2 |  |  |  |  |  |
| 5.93 | PHP-3 | PHP-3 | PHP-3 |  |  |  |  |  |
| 5.97 | PHP-3 | PHP-3 | PHP-3 |  |  |  |  |  |
| 6.01 | - | PHP-3 | PHP-3 |  |  |  |  |  |
| 6.05 | AMD-2 | AMD-2 | AMD-2 |  |  |  |  |  |
| 6.09 | ALK-1 | ALK-1 | - |  |  |  |  |  |
| 6.13 | ALK-1 | ALK-1 | STR-1 |  |  |  |  |  |
| 6.17 | PHP-1 | PHP-1 | PHP-1 |  |  |  |  |  |
| 6.21 | PHP-1 | PHP-1 | PHP-1 |  |  |  |  |  |
| 6.25 | FLV-1 | FLV-1 | FLV-2 |  |  |  |  |  |
| 6.29 | FLV-2 | FRC-2 | FRC-2 |  |  |  |  |  |
| 6.33 | IRG-2 | IRG-2 | IRG-1 |  |  |  |  |  |
| 6.37 | IRG-3 | FRC-2 | FLV-2 |  |  |  |  |  |
| 6.41 | IRG-1 | FRC-1 | FRC-2 |  |  |  |  |  |
| 6.45 | FLV-1 | FLV-1 | FLV-1 |  |  |  |  |  |
| 6.49 | PHP-1 | PHP-1 | PHP-1 |  |  |  |  |  |
| 6.53 | PHP-1 | PHP-1 | PHP-1 |  |  |  |  |  |
| 6.57 | ALK-2 | ALK-2 | ALK-2 |  |  |  |  |  |
| 6.61 | PHP-3 | PHP-3 | PHP-3 |  |  |  |  |  |
| 6.65 | PHP-3 | PHP-3 | PHP-3 |  |  |  |  |  |
| 6.69 | PHP-3 | PHP-3 | PHP-3 |  |  |  |  |  |
| 6.73 | PHP-3 | PHP-3 | PHP-3 |  |  |  |  |  |
| 6.77 | PHP-3 | PHP-3 | PHP-3 |  |  |  |  |  |
| 6.81 | PHP-1 | PHP-1 | PHP-1 |  |  |  |  |  |
| 6.85 | - | PHP-1 | PHP-1 |  |  |  |  |  |
| 6.89 | - | FLV-2 | - |  |  |  |  |  |
| 6.93 | FLV-2 | FLV-2 | AMD-2 |  |  |  |  |  |
| 6.97 | PHP-1 | PHP-1 | PHP-1 |  |  |  |  |  |
| 7.01 | - | PHP-1 | PHP-1 |  |  |  |  |  |
| 7.05 | AMD-1 | PHP-1 | AMD-1 |  |  |  |  |  |
| 7.09 | AMD-2 | AMD-2 | AMD-2 |  |  |  |  |  |
| 7.13 | AMD-2 | FRC-2 | FRC-2 |  |  |  |  |  |
| 7.17 | - | FRC-2 | FRC-2 |  |  |  |  |  |
| 7.21 | FLV-3 | FRC-2 | FRC-2 |  |  |  |  |  |
| 7.25 | FLV-3 | FLV-3 | FRC-2 |  |  |  |  |  |
| 7.29 | FLV-2 | FLV-2 | FLV-3 |  |  |  |  |  |
| 7.33 | AMD-2 | AMD-2 | AMD-2 |  |  |  |  |  |
| 7.37 | PHP-1 | PHP-1 | PHP-1 |  |  |  |  |  |
| 7.41 | FLV-3 | PHP-1 | FLV-2 |  |  |  |  |  |
| 7.45 | FLV-2 | FLV-2 | FLV-2 |  |  |  |  |  |
| 7.49 | FLV-2 | FLV-2 | FRC-2 |  |  |  |  |  |
| 7.53 | IRG-1 | FRC-2 | FRC-2 |  |  |  |  |  |
| 7.57 | IRG-1 | FRC-1 | - |  |  |  |  |  |
| 7.61 | AMD-2 | FRC-1 | - |  |  |  |  |  |
| 7.65 | AMD-2 | FLV-1 | AMD-2 |  |  |  |  |  |
| 7.69 | FLV-1 | FLV-1 | FLV-1 |  |  |  |  |  |
| 7.73 | FLV-1 | FRC-2 | FRC-2 |  |  |  |  |  |
| 7.77 | ALK-3 | ALK-3 | ALK-3 |  |  |  |  |  |
| 7.81 | STR-3 | FRC-2 | FRC-2 |  |  |  |  |  |
| 7.85 | - | FRC-2 | FRC-2 |  |  |  |  |  |
| 7.89 | STR-1 | FRC-1 | - |  |  |  |  |  |
| 7.93 | IRG-1 | FRC-1 | IRG-1 |  |  |  |  |  |
| 7.97 | IRG-1 | IRG-3 | FRC-2 |  |  |  |  |  |
| 8.01 | ALK-2 | ALK-2 | FRC-2 |  |  |  |  |  |
| 8.05 | FLV-3 | FRC-1 | - |  |  |  |  |  |
| 8.09 | FLV-2 | FLV-2 | FLV-2 |  |  |  |  |  |
| 8.13 | FLV-3 | FLV-2 | FLV-2 |  |  |  |  |  |
| 8.17 | FLV-3 | FLV-2 | FLV-3 |  |  |  |  |  |
| 8.21 | FLV-2 | FLV-2 | FLV-3 |  |  |  |  |  |
| 8.25 | FLV-3 | FRC-2 | FRC-2 |  |  |  |  |  |
| 8.29 | FLV-2 | FLV-3 | FRC-2 |  |  |  |  |  |
| 8.33 | FLV-2 | FLV-3 | FRC-2 |  |  |  |  |  |
| 8.37 | FLV-2 | FLV-2 | - |  |  |  |  |  |
| 8.41 | - | - | - |  |  |  |  |  |
| 8.45 | FLV-2 | FLV-2 | - |  |  |  |  |  |
| 8.49 | FLV-3 | FRC-2 | - |  |  |  |  |  |
| 8.53 | - | - | - |  |  |  |  |  |
| 8.57 | IRG-2 | IRG-2 | IRG-1 |  |  |  |  |  |
| 8.61 | - | - | - |  |  |  |  |  |
| 8.65 | - | - | - |  |  |  |  |  |
| 8.69 | - | - | - |  |  |  |  |  |
| 8.73 | - | - | - |  |  |  |  |  |
| 8.77 | - | - | - |  |  |  |  |  |
| 8.81 | - | - | - |  |  |  |  |  |
| 8.85 | - | - | - |  |  |  |  |  |
| 8.89 | - | - | - |  |  |  |  |  |
| 8.93 | - | - | - |  |  |  |  |  |
| 8.97 | FLV-3 | ALK-2 | - |  |  |  |  |  |
| 9.01 | - | - | - |  |  |  |  |  |
| 9.05 | PHP-1 | - | - |  |  |  |  |  |
| 9.09 | PHP-1 | - | - |  |  |  |  |  |
| 9.13 | PHP-1 | PHP-4 | - |  |  |  |  |  |
| 9.16 | - | PHP-4 | - |  |  |  |  |  |
| 9.20 | PHP-1 | - | - |  |  |  |  |  |
| 9.24 | - | - | - |  |  |  |  |  |
| 9.28 | PHP-1 | - | - |  |  |  |  |  |
| 9.32 | - | PHP-4 | - |  |  |  |  |  |
| 9.36 | - | - | - |  |  |  |  |  |
| 9.40 | - | FRC-2 | FRC-2 |  |  |  |  |  |
| 9.44 | - | - | - |  |  |  |  |  |
| 9.48 | PHP-1 | - | STR-1 |  |  |  |  |  |
| 9.52 | - | - | - |  |  |  |  |  |
| 9.56 | - | - | - |  |  |  |  |  |
| 9.60 | - | PHP-3 | - |  |  |  |  |  |
| 9.64 | FLV-1 | - | - |  |  |  |  |  |
| 9.68 | - | - | - |  |  |  |  |  |
| 9.72 | - | - | - |  |  |  |  |  |
| 9.76 | PHP-2 | PHP-2 | - |  |  |  |  |  |
| 9.80 | PHP-2 | PHP-2 | - |  |  |  |  |  |
| 9.84 | - | - | - |  |  |  |  |  |
| 9.88 | - | - | - |  |  |  |  |  |
| 9.92 | - | TPN-3 | - |  |  |  |  |  |
| 9.96 | TPN-3 | TPN-3 | TPN-3 |  |  |  |  |  |
| 10.00 | TPN-3 | TPN-3 | - |  |  |  |  |  |
| 10.04 | - | - | - |  |  |  |  |  |
| 10.08 | - | - | - |  |  |  |  |  |
| 10.12 | - | - | - |  |  |  |  |  |
| 10.16 | - | - | - |  |  |  |  |  |
| 10.20 | - | - | - |  |  |  |  |  |
| 10.24 | - | - | - |  |  |  |  |  |
| 10.28 | - | - | - |  |  |  |  |  |
| 10.32 | - | - | - |  |  |  |  |  |
| 10.36 | - | - | - |  |  |  |  |  |
| 10.40 | - | - | - |  |  |  |  |  |
| 10.44 | - | - | - |  |  |  |  |  |
| 10.48 | - | - | - |  |  |  |  |  |
| 10.52 | - | - | - |  |  |  |  |  |
| 10.56 | - | - | - |  |  |  |  |  |
| 10.60 | - | - | - |  |  |  |  |  |
| 10.64 | - | - | - |  |  |  |  |  |
| 10.68 | IRG-2 | - | - |  |  |  |  |  |
| 10.72 | - | - | - |  |  |  |  |  |
| 10.76 | - | - | - |  |  |  |  |  |
| 10.80 | - | - | - |  |  |  |  |  |
| 10.84 | - | - | - |  |  |  |  |  |
| 10.88 | - | - | - |  |  |  |  |  |
| 10.92 | - | - | - |  |  |  |  |  |
| 10.96 | IRG-2 | - | - |  |  |  |  |  |
| 11.00 | IRG-2 | - | - |  |  |  |  |  |
| 11.04 | - | - | - |  |  |  |  |  |
| 11.08 | - | - | - |  |  |  |  |  |
| 11.12 | - | - | - |  |  |  |  |  |
| 11.16 | - | - | - |  |  |  |  |  |
| 11.20 | - | - | - |  |  |  |  |  |
| 11.24 | - | - | - |  |  |  |  |  |
| 11.28 | IRG-2 | - | - |  |  |  |  |  |
| 11.32 | - | - | - |  |  |  |  |  |
| 11.36 | - | - | - |  |  |  |  |  |
| 11.40 | - | - | - |  |  |  |  |  |
| 11.44 | - | - | - |  |  |  |  |  |
| 11.48 | - | - | - |  |  |  |  |  |
| 11.52 | - | - | - |  |  |  |  |  |
| 11.56 | - | - | - |  |  |  |  |  |
| 11.60 | - | - | - |  |  |  |  |  |
| 11.64 | - | - | - |  |  |  |  |  |
| 11.68 | - | - | - |  |  |  |  |  |
| 11.72 | - | - | - |  |  |  |  |  |
| 11.76 | - | - | - |  |  |  |  |  |
| 11.80 | - | - | - |  |  |  |  |  |
| 11.84 | IRG-2 | - | - |  |  |  |  |  |
| 11.88 | IRG-2 | - | - |  |  |  |  |  |
| 11.92 | - | - | - |  |  |  |  |  |
| 11.96 | - | - | - |  |  |  |  |  |
| 12.00 | - | - | - |  |  |  |  |  |
