# Supplementary material for: Shedding Light on Chemically Mediated Tri-Trophic Interactions: A 1H-NMR Network Approach to Identify Compound Structural Features and Associated Biological Activity
Source: Front Plant Sci. 2018 Aug 17;9:1155. doi: 10.3389/fpls.2018.01155 (PMC6107749; doi:10.3389/fpls.2018.01155)
Supplement: Supplementary file 6 [file Table_6.DOCX]

Table S6. Structural features described by the modules, accordingly to the unified code.

| Module | Description |
| --- | --- |
| ALK-1 | Olefin in the β-position to heteroatoms (especialy nitrogen) |
| ALK-2 | Aromatic alkaloids: α-position to nitrogen, in addition to aromatic and methoxy signals |
| ALK-3 | Allyl ether or amine, in addition to α-position to nitrogen |
| AMD-1 | Dihydro-pyridinone and its epoxide form; also contains elements of the hydrocinnamyl moiety. |
| AMD-2 | α-position to nitrogen and other elements of the dihydro-pyridinone ring; it also contains elements of the cinnamyl moiety |
| FLV-1 | Glucosylated isoflavone (oxygenated and aromatic methines) |
| FLV-2 | Flavone (aromatic peaks) |
| FLV-3 | Glucosylated flavones |
| FRC-1 | Furanocoumarins oxygenated in position 8, with peaks for prenyl group. |
| FRC-2 | Furanocoumarins oxygenated in position 5. |
| GLC-1 | Sugar moieties |
| IRG-1 | Oxydated iridoid glycosides: emphasis on pyran ring and glucose moiety. Aromatic peaks are specific to catapolside. |
| IRG-2 | Oxydated iridoid glycosides: emphasis on pyran ring and glucose moiety (inclusively anomeric position). |
| IRG-3 | Unsaturated iridoid glycosides (olefins and glucose moiety) |
| IRG-4 | Pyran ring (olefin methine in α-position to oxygen) |
| PHP-1 | Oxygenated stilbenoid |
| PHP-2 | Prenylated aryl with high degree of oxydation |
| PHP-3 | Oxygenated phenylpropene |
| PHP-4 | Phenolic peaks, specific to resveratrol in the presence of Escin |
| STR-1 | Exclusive to escin |
| STR-2 | General elements of triterpene structures, with some emphasis on oxydated positions and allyl methyls. |
| STR-3 | Triterpenoid structures with oxydation on ring A, aliphatic methyl groups, and unsaturated ring (B or C). |
| TPN-1 | Allyl methyls, aliphatic vinyl methine or terminal alkenes |
| TPN-2 | Allyl methylenes, deshielded vinyl methines |
| TPN-3 | Saturated aliphatic structures with ramifications (methyls), and allyl methanol |
