# Supplementary material for: Shedding Light on Chemically Mediated Tri-Trophic Interactions: A 1H-NMR Network Approach to Identify Compound Structural Features and Associated Biological Activity
Source: Front Plant Sci. 2018 Aug 17;9:1155. doi: 10.3389/fpls.2018.01155 (PMC6107749; doi:10.3389/fpls.2018.01155)
Supplement: Supplementary file 7 [file Table_7.DOCX]

Table S7. Summary statistics and generalized model results comparing multiple to single species analysis.

|  | Multiple *Piper* species | *Piper kelleyi* | t | p-value | AIC |
| --- | --- | --- | --- | --- | --- |
| Average Module Eigenvalue | 0.082 (± 0.006) | 0.110 (±0.005) | 3.369 | 0.001 | -401.03 |
| Average Module Variation | 0.097 (± 0.006) | 0.085 (±0.005) | -1.565 | 0.12 | -407.31 |
| Module Count | 10.45 (± 0.51) | 7.19 (±0.40) | -4.664 | < 0.001 | 578.78 |
