# Supplementary material for: Shedding Light on Chemically Mediated Tri-Trophic Interactions: A 1H-NMR Network Approach to Identify Compound Structural Features and Associated Biological Activity
Source: Front Plant Sci. 2018 Aug 17;9:1155. doi: 10.3389/fpls.2018.01155 (PMC6107749; doi:10.3389/fpls.2018.01155)
Supplement: Supplementary file 8 [file Data_Sheet_1.DOCX]

Sample R code, see <https://labs.genetics.ucla.edu/horvath/CoexpressionNetwork/Rpackages/WGCNA/> for more package details.

##install the package#

source("http://bioconductor.org/biocLite.R")

biocLite(c("AnnotationDbi", "impute", "GO.db", "preprocessCore"))

install.packages("WGCNA")

install.packages("tibble")

library('WGCNA')

#read in data, each row is a sample and each column is a binned ppm in NMR#

mixpip<-read.table("my_data", header = TRUE, row.names = 1)

#create a plot to determine your threshold, this may be all over the place we start with the threshold at the beginning of the plateau#

powers = c(c(1:10), seq(from = 12, to=20, by=2))

sft = pickSoftThreshold(mixpip, powerVector = powers )

plot(sft$fitIndices[,1], -sign(sft$fitIndices[,3])*sft$fitIndices[,2],

xlab="Soft Threshold (power)",ylab="Scale Free Topology Model Fit,signed R^2",type="n",

main = paste("Scale independence"));

text(sft$fitIndices[,1], -sign(sft$fitIndices[,3])*sft$fitIndices[,2],

labels=powers, col="red");

#calculated modules using the threshold you determined from plot, set it as power = #

netpip= blockwiseModules(mixpip, power =11,

TOMType = "unsigned", minModuleSize =3,

reassignThreshold = 0, mergeCutHeight = 0.25,

numericLabels = TRUE, pamRespectsDendro = FALSE,

saveTOMs = TRUE,

saveTOMFileBase = "C:/R/mixoutTOM.csv")

#this will give you a list of the modules and the number of nodes in them#

table(netpip$colors)

#merge the color names with module number and plot dendrogram#

mergedColors = labels2colors(netpip$colors)

plotDendroAndColors(netpip$dendrograms[[1]], mergedColors[netpip$blockGenes[[1]]],

"Module colors",

dendroLabels = FALSE, hang = 0.03,

addGuide = TRUE, guideHang = 0.05)

#prep data for export and other analyses#

moduleLabels = netpip$colors

moduleColors = labels2colors(netpip$colors)

MEs = netpip$MEs;

geneTree = netpip$dendrograms[[1]];

save(MEs, moduleLabels, moduleColors, geneTree,

file = "networkConstruction-auto.RData")

lnames = load(file = "networkConstruction-auto.RData");

MEs0 = moduleEigengenes(mixpip, moduleColors)$eigengenes

MEs = orderMEs(MEs0)

lnames

nGenes = ncol(mixpip);

nSamples = nrow(mixpip);

sizeGrWindow(6,6)

par(cex=1.0)

plotEigengeneNetworks(MEs, "Module dendrogram", marDendro = c(0,4,1,0), plotHeatmaps=FALSE)

plotEigengeneNetworks(MEs, "Module adjacency heatmap", marHeatmap = c(3,4,2,2), plotDendrograms = FALSE, xLablesAngle=90)

write.csv(MEs,"C:/R/ME.csv") # gives a file of modules and eigenvalues across samples, samples are just numbered

write.csv(moduleColors,"C:/R/modules.cvs")# gives a file of list the chemical shift (node) for each module

#To make the heat map of modules to ecological or biological data#

#In this analysis we ran the module analysis with both crude extracts and prepared mixtures.

#We separated the ME files so we can run separate analyses, for simplicity we are reading in a new ME file

pip2<-read.csv("C:/R/ME.csv",header=T, row.names = 1)

#I read in the biological data table with samples in rows ordered exactly same as the chemistry data

biol<-read.table("C:/R/GiftLSmixbio.txt", header = TRUE, row.names = 1)

#module to biological data

moduleCor = cor(pip2, biol, use = "p");

modulePvalue = corPvalueStudent(moduleCor, nSamples);

textMatrix = paste(signif(moduleCor, 2), "\n(",

signif(modulePvalue, 1), ")", sep = "");

dim(textMatrix) = dim(moduleCor)

par(mar = c(6, 8.5, 3, 3));

labeledHeatmap(Matrix = moduleCor,

xLabels = names(biol),

yLabels = names(pip2),

ySymbols = names(pip2),

colorLabels = FALSE,

colors = blueWhiteRed(50),

textMatrix = textMatrix,

setStdMargins = TRUE, cex.text = 0.65, zlim = c(-1,1),

main = paste("Module-biological activity relationships"))

#Exporting data for network figure

options(stringsAsFactors = FALSE);

#recaclulate to Topological overlap matrix;

TOM=TOMsimilarityFromExpr(mixpip, power=11)

#Specify modules to include, otherwise it will include all the modules,

modules = c( "blue","darkred", "grey60","yellow","purple","turquoise","pink","magenta","brown","lightcyan","salmon","darkgreen","lightyellow","green","tan","lightgreen","midnightblue","cyan","darkturquoise","black","royalblue","greenyellow","red");

probes = names(mixpip)

inModule = is.finite(match(moduleColors, modules));

modProbes = probes[inModule];

modTOM = TOM[inModule, inModule];

dimnames(modTOM) = list(modProbes, modProbes)

#export the edge and nodes file for cytoscape

cyt = exportNetworkToCytoscape(modTOM,

edgeFile = paste("C:/R/Cyto-edges", ".txt", sep=""),

nodeFile = paste("C:/R/Cyto-nodes", ".txt", sep=""),

weighted = TRUE,

threshold = 0.02,

nodeNames = modProbes,

altNodeNames = modules,

nodeAttr = moduleColors[inModule])
